# Comparative functional RNA editomes of neural differentiation from human PSCs

**DOI:** 10.1093/lifemedi/lnac027

**Published:** 2022-08-10

**Authors:** Yu Zhang, Qu Zhang, Yuhong Hou, Ran Wang, Yu Wang

**Affiliations:** College of Life Sciences and Oceanography, Shenzhen University, Shenzhen 518055, China; Mlobio, Singularity Center, Beijing 102200, China; College of Life Sciences and Oceanography, Shenzhen University, Shenzhen 518055, China; Experimental Medicine Unit, GlaxoSmithKline, Collegeville, PA 19426, USA; State Key Laboratory of Stem Cell and Reproductive Biology, Institute of Zoology, Chinese Academy of Sciences, Beijing 100101, China; University of Chinese Academy of Sciences, Beijing 100049, China; Cell Resource Center, Peking Union Medical College (PUMC), Beijing 100005, China; State Key Laboratory of Stem Cell and Reproductive Biology, Institute of Zoology, Chinese Academy of Sciences, Beijing 100101, China; University of Chinese Academy of Sciences, Beijing 100049, China; Peking Union Medical College Hospital, Beijing 100730, China; College of Life Sciences and Oceanography, Shenzhen University, Shenzhen 518055, China

**Keywords:** RNA editing, CRISPR/Cas9, human pluripotent stem cell, neural differentiation, ZYG11b

## Abstract

RNA editing is a fundamental mechanism that constitutes the epitranscriptomic complexity. A-to-G editing is the predominant type catalyzed by ADAR1 and ADAR2 in human. Using a CRISPR/Cas9 approach to knockout *ADAR1/2*, we identified a regulatory role of RNA editing in directed differentiation of human embryonic stem cells (hESCs) toward neural progenitor cells (NPCs). Genome-wide landscapes of A-to-G editing in hESCs and four derivative cell lineages representing all three germ layers and the extraembryonic cell fate were profiled, with a particular focus on neural differentiation. Furthermore, a bioinformatics-guided case study identified a potential functional editing event in *ZYG11B* 3ʹUTR that might play a role in regulation of NPC differentiation through gain of *miR6089* targeting. Collectively, our study established the functional role of A-to-G RNA editing in neural lineage differentiation; illustrated the RNA editing landscapes of hESCs and NPC differentiation; and shed new light on molecular insights thereof.

## Introduction

RNA editing is an epitranscriptional mechanism that introduces changes in RNA sequences, an important component constituting the complexity of biological circuits [1, 2]. Among over 100 different types of RNA modifications, N6-methyladenosine (m6A), the most abundant, gained dominant limelight, while others, including editing, remain largely unexplored [3]. Adenosine-to-inosine editing in double-stranded RNA (dsRNA) substrates is the most common type of editing and the second abundant RNA modification, just short of m6A. It is catalyzed by the adenosine deaminase acting on RNA (ADAR) enzymes [4–6]. Inosine is recognized as Guanosine (G) upon translation or reverse transcription of RNA molecules. A-to-G editing occurs predominantly within Alu repetitive elements possibly due to their double-stranded feature. Alu repeats exist specifically in primates, thus resulting in dramatically expanded RNA editing. Such distinction from other organisms was proposed to be a potential driving force for increased cognition and neural evolution [7]. There are three *ADAR* homologs in mammals, *ADAR1 (ADAR), ADAR2 (ADARB1),* and *ADAR3 (ADARB2)*. ADAR1 and ADAR2 are responsible for A-to-G editing while ADAR3 having no editase activity and rather inhibitory potentially through competing for dsRNA substrates [8]. A much rarer type of RNA editing, cytidine-to-uridine, is mediated by the APOBEC (apolipoprotein B mRNA-editing enzyme, catalytic polypeptide-like) enzymes [9]. RNA editing modulates the activity of a substrate transcript in diverse mechanisms, including recoding a protein, regulating its stability or subcellular localization, and redirecting microRNA (miRNA) targeting if editing occurs in a miRNA or its target [5]. Aberrant RNA editing has been linked with a variety of diseases, primarily neurological disorders, autoimmune diseases, and cancers [10, 11].

Pluripotent stem cells (PSCs) and their differentiation toward different cell types in distinct germ layers underline central processes governing human embryonic development. Human pluripotent stem cells (hPSCs), including human embryonic stem cells (hESCs) and induced pluripotent stem cells, and their directed differentiation *in vitro* provide a model system for studying these processes, which is also of enormous therapeutic potential as a source to produce functional cells for drug testing and cell therapy [12–14].

Earlier studies suggest an essential role of A-to-G editing in embryonic development in mammals. Genetic ablation of *Adar1* in mouse resulted in lethal defects during embryonic development [15–18]. In addition, several lines of evidences suggest A-to-G RNA editing might play a role in maintaining hPSCs and lineage differentiation: overexpression of ADAR1-p110, the isoform that is constitutively expressed, is readily achievable in a differentiated cell type but cannot be achieved in hESCs [19]; conversely, RNAi mediated down-regulation of ADAR1 leads to up-regulation of genes involved in differentiation [20]. Moreover, RNA editing is enriched in human brain. Dysregulation of RNA editing is involved in a multitude of neurological and psychiatric disorders, including schizophrenia, bipolar disease, autism, epilepsy, and amyotrophic lateral sclerosis [21–24]. The editing level of the glutamate receptor GluA2 Q/R site might be altered in epilepsy, amyotrophic lateral sclerosis, and schizophrenia [25, 26]. Dysregulated 5-HT2CR (HTR2C) RNA editing might be involved in depression and suicide, schizophrenia, and Prader-Willi syndrome [27, 28]. More recently, large cohort studies reveal widespread altered RNA editing in schizophrenia and autism, and suggest a causal role of RNA editing in these disorders [29, 30]. Despite of reported roles for ADAR1 function independent of editing, such as miRNA processing, highly similar phenotypes derived from *ADAR1* null and editing-deficient *ADAR1 E861A* mutant alleles strongly argues against important function beyond editing [31–34]. Therefore, whether A-to-G editing plays a functional role in hPSCs and neural differentiation remains elusive.

Here we first present the evidence of a regulatory role of ADAR1 in the neural lineage differentiation from hESCs. Meanwhile, A-to-G RNA editing appears to be dispensible in hESCs. We then profiled RNA editomes for human pluripotent state and multilineage differentiation, with RNA-Seq data from a human ES cell line H1 and four cell lineages derived from H1, including mesendoderm (ME), mesenchymal stem cell (MSC), neural progenitor cell (NPC), and trophoblast-like cell (TBL), representing cell fates of all three germ layers and extraembryonic commitment [35]. We further focused on neural lineage differentiation and identified a potential functional editing site in *ZYG11B* 3ʹUTR, whose editing leads to gain of *miR6089* targeting, thus a decreased mRNA level. Through CRISPR/Cas9 mediated disruption of *miR6089* seed sequence, we provided evidences supporting that *miR6089* might play a role in regulating NPC differentiation through modulating ZYG11B mRNA level.

## Results

### Functional investigations of A-to-G editing in hESC maintenance and NPC differentiation

**Figure 1.**
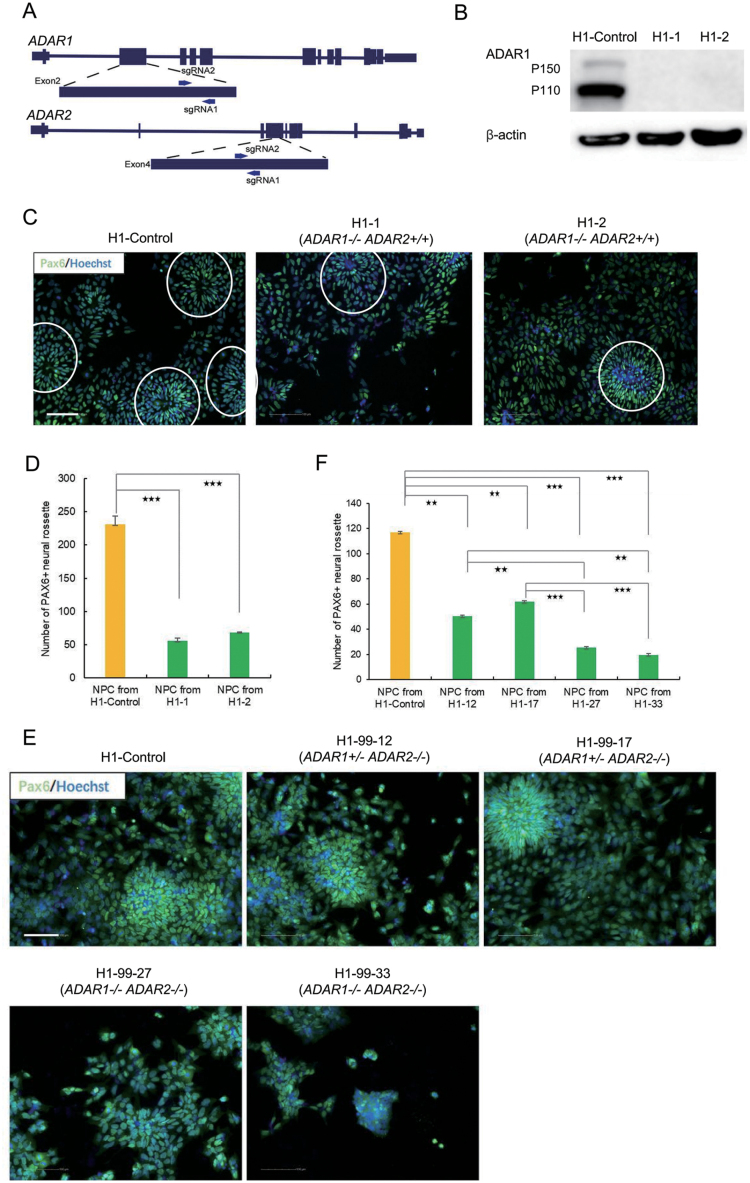
Generation and characterization of ***ADAR1/2*** knockout H1 cell lines. (A) Schematics showing *ADAR1/2* loci and sgRNA target sites. (B) Western blotting confirmed *ADAR1* genotypes in H1 cell lines. A wild-type monoclonal H1 cell line (H1-control) generated from the same round of CRISPR/Cas9 genome editing was used as a control. Actin was used as a loading control. (C–D) Representative images of Pax6 (green) and Hoechst (blue) (C) and quantitative analyses (D) of two *ADAR1*^*−/−*^ monoclonal H1 cell lines (H1-1 and H1-2), in comparison with a wild-type H1 cell line (H1-control) generated from the same round of CRISPR/Cas9 genome editing. Pax6+ neural rosettes were labeled with circles. (E–F) Representative images of Pax6 (green) and Hoechst (blue) (E) and quantitative analyses (F) of two *ADAR1*^*−/−*^ *ADAR2*^*−/−*^ monoclonal H1 cell lines (H1-99-27 and H1-99-33), in comparison with a wild-type H1 cell line (H1-control) and two *ADAR1*^*+/−*^ *ADAR2*^*−/−*^ monoclonal clones (H1-99-12 and H1-99-17) generated from the same round of CRISPR/Cas9 genome editing. Scale bar, 100 µm. Data are presented as mean ± SD. *n* = 3 Student *t*-test, (**) *P* < 0.01, (***) *P* < 0.001.

First, we sought to completely abolish A-to-G editing in H1 cells using the clustered, regularly interspaced, short palindromic repeats (CRISPR)/CRISPR associated protein 9 (Cas9). Two single guide RNAs (sgRNA) targeting 5ʹ sequence in the coding regions of *ADAR1* and *ADAR2* respectively were co-delivered to H1 cells with Cas9 (Fig. 1A). Monoclonal cell lines were screened for *ADAR1/2* knockout through frame shift and/or premature stop codons introduced by CRISPR/Cas9 mediated nonhomologous end-joining (NHEJ) events. Targeted amplification and Sanger sequencing of *ADAR1/2* alleles were conducted to identify the genotypes of each monoclone (data not shown). We first examined *ADAR1*^*−/−*^*; ADAR2*^*+/+*^ H1 cell lines (H1-1 and H1-2). Western blot analyses of ADAR1 protein expression confirmed their genotypes identified from DNA sequencing (Fig. 1B). No noticeable change in cellular proliferation or pluripotent marker expression was observed (Figs. S1A and S1B). Given the importance of RNA editing in neural system, we next examined whether directed neural differentiation from H1 is affected upon *ADAR1* knockout. Using Pax6 as a specific marker of NPCs, which form neural rosettes [36], we found that directed differentiation to Pax6+ neural rosettes was significantly impaired in *ADAR1*^*−/−*^*; ADAR2*^*+/+*^ H1 cells (Figs. 1C and 1D). A control H1 cell line harvested simultaneously but without sequence alteration in *ADAR1* or *ADAR2* loci was used for comparison (Figs. 1C and 1D). Doxycycline inducible RNAi using two short hairpin RNAs (shRNAs) targeting ADAR1 also lead to less efficient NPC differentiation and such effect could be rescued by overexpression of ADAR1 coding sequence in which the shRNA target regions are recoded with synonymous codons (Fig. S2). These data suggest that ADAR1 plays a role in the regulation of NPC differentiation. We also examined directed differentiation toward TBL and ME respectively, neither of which showed consistent alteration upon ADAR knockout (Figs. S3 and S4). This implies that the role of ADAR1 in differentiation toward the neural fate might be specific. Nonetheless, the functional role of A-to-G editing in ESC maintenance remained to be determined, as previous observation using *ADAR1* knockout cell lines could not rule out a redundant compensation from *ADAR2*. Unfortunately, no homozygous compound knockout was obtained from our initial attempts of genome targeting. This might be due to the low efficiency to introduce NHEJ events in four alleles of *ADARs* simultaneously. To this end, we used an *ADAR1*^*+/−*^*; ADAR2*^*−/−*^ ESC line (H1-99) generated from the initial rounds of CRISPR/Cas9 genome editing and repeatedly delivered constructs to target the single wild-type allele of *ADAR1*. As a result, *ADAR1*^*−/−*^*; ADAR2*^*−/−*^ compound knockout cell lines (H1-99-27 and H1-99-33) were obtained. Western blot analyses validated complete knockout of *ADAR1* following DNA sequencing, although we could not identify a qualified antibody for ADAR2 examination (Fig. S5A). We also included in our analyses other monoclonal cell lines (H1-99-12 and H1-99-17) harvested simultaneously but without sequence alteration in *ADAR1* or *ADAR2* loci and found that ADAR1 protein level was obviously decreased, consistent with their heterozygous *ADAR1* alleles (Fig. S5A). Cell proliferation and pluripotency markers were still not altered in these *ADAR1*^*−/−*^*; ADAR2*^*−/−*^ compound knockout cell lines (Figs. S5B and S5C), thus clarifying that A-to-G editing mediated by ADARs is dispensable for hPSC maintenance. Directed differentiation to NPC rosettes was further impaired in comparison with monoclones of an *ADAR1*^*+/−*^*; ADAR2*^*−/−*^ genotype harvested simultaneously (Figs. 1E and 1F), thus suggesting a dose-dependent role of ADAR1 in regulation of NPC differentiation. In sum, genetic ablation of *ADARs* using a CRISPR/Cas9 approach demonstrated an important functional role of A-to-G editing in NPC differentiation.

### Refinement of bioinformatics pipeline to identify RNA editing sites in hESCs

**Figure 2.**
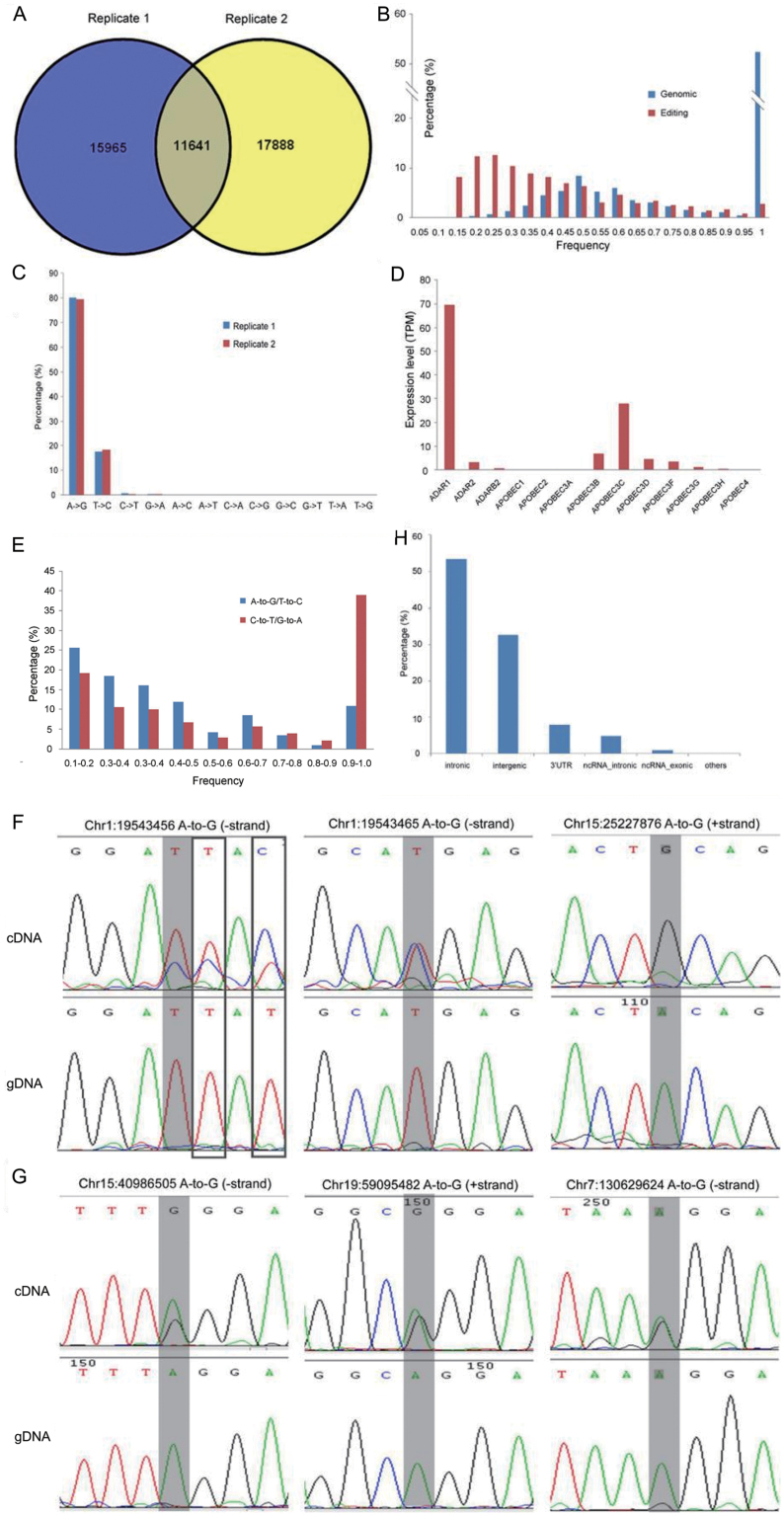
Identification of editing sites in H1, expression of editing enzymes, and validation of predicted A-to-G (or T-to-C on the strand) editing sites by Sanger sequencing. (A) 11641 editing sites were identified in both replicates of H1 RNA-Seq and were included in the following analyses. (B) The frequency distribution of RNA editing sites (red) and genomic variants found in dbSNP or 1000 Genomes database (blue). (C) Distribution of each substitution type in two replicates of H1 RNA-Seq. (D) The expression abundance of enzymes involved in RNA editing process (in transcript per million reads estimated using RSEM). (E) The frequency distribution of the less common editing type C-to-T in comparison to A-to-G editing. (F–G) Editing sites in coding genes (F) and ncRNAs (G). The editing positions were highlighted by gray shading. Top and bottom panels are results from cDNA and genomic DNA respectively. False negatives were highlighted in boxes. (H) Genomic distribution of editing sites.

Next we sought to profile RNA editing events in H1 and NPC on a genomic scale. First, data generated from two replicate samples of the H1 cell line were used to tune the data analysis pipeline. RNA editing sites in each replicate were identified separately at this stage. Paired-end reads of 101 bp of DNA-Seq and RNA-Seq were mapped to human reference genome and a collection of splicing junctions respectively. A series of filters including that for PCR duplicates were applied to identify potential *bona fide* editing sites and to avoid false positives. As a result, we identified 27,606 and 29,529 putative editing sites in replicate 1 and replicate 2 respectively, and 11,641 editing sites in both replicates (Fig. 2A). In contrast to replicate-specific sites, shared sites correlate with significantly more read depth (14 vs 7, median read depth, *P*-value < 0.001, Wilcoxon ranksum test). Therefore, we only considered sites of high confidence found in both replicates, though it is possible that some replicate-specific editing sites are true positives and were undetectable in the other replicate due to the stochastic feature of the sequencing process. Consistent with previous observations, most sites (~97%) are in Alu regions. The frequency distribution of editing sites shows a distinct pattern in comparison with that of genomic variants found in dbSNP databases (Fig. 2B). Among the distinct types of substitutions, we discovered that A-to-G editing is most prevalent, accounting for ~80% of all editing sites (Fig. 2C). The second most abundant is T-to-C substitution (~18%), which is complementary to A-to-G editing. Given that we assigned the strand information for editing sites solely using available annotations, it is possible T-to-C substitutions are actually A-to-G changes, but were assigned to the opposite strand. In fact, approximately two-third of T-to-C substitutions were in regions with no annotation. But when interrogating T-to-C sites in the DARNED database [37], we found that the substitution state is A-to-G on the opposite strand for every T-to-C site overlapping with a DARNED editing position. We thus concluded that most, if not all, of T-to-C substitutions in our list were virtually A-to-G on the opposite strand. This resulted in ~98% A-to-G sites in total, consistent with previous reports [38–40]. As a result, we obtained 11,452 high-quality A-to-G editing sites in H1, much more than 4151 sites in H1 from a latest report that were identified through alignments with known editing sites [41] (Supplementary File 2). This also indicates that a substantial portion of the editing sites we identified herein were novel.

Although the current study focuses on A-to-G editing, we also conducted preliminary analyses on C-to-T (U) editing, a much rarer type of editing catalyzed by APOBEC family [9]. We observed that C-to-T is the second most prevalent editing event, assuming that, for the reasons discussed above, G-to-A editing is counted as C-to-T. Several members of the APOBEC family are expressed at comparable levels to the *ADAR* genes (Fig. 2D). We examined the distribution of editing frequency for the A-to-G (and T-to-C) and the C-to-T (and G-to-A) sites and found that the C-to-T (and G-to-A) sites are significantly skewed to very high editing level (*P*-value < 2.2 × 10^−16^, chi-square test, Fig. 2E), possibly due to editing activities of APOBEC on both RNA and DNA.

We next applied PCR and Sanger sequencing to examine predicted editing events. Out of 26 randomly selected putative A-to-G editing events in both coding RNAs (Figs. 2F and S6A) and long noncoding RNAs (Figs. 2G and S6B), 77% were validated by Sanger sequencing. Since different batches of H1 cells were used for validation, we may expect some discrepancy of editing events and a validation rate of 77% suggests most of the editing events identified herein are real. On the contrary, none of four “non-canonical” editing events can be detected in paralleled examinations (Fig. S6C), thus lending more support to lack of “non-canonical” editing mechanisms [42]. In addition, our experiments also identified false negatives missed by the pipeline possibly due to the use of strict filters (Fig. 2F, boxed). Assuming that all other changes are considered as errors, the false positive rate is ~7%. Collectively, our pipeline is sufficient to identify *bona fide* A-to-G editing sites.

An important question is whether the editing events are conserved in other hPSC lines. Accordingly, we cross-examined a number of editing sites in another hES cell line H9 [13] and a hiPSC line IMR90-4 [14]. Out of 25 A-to-G editing sites examined, editing of 23 sites was also observed in both H9 and IMR90-4 (Figs. S7A and S7B). No non-canonical editing events were observed in H9 or IMR90-4, thus further arguing against the existence of such mechanisms (Fig. S7C). Having experimentally validated these editing events, we also comparatively analyzed editing level of 6 randomly selected sites in H1 cell lines of distinct *ADAR1/2* genotypes, including wildtype, *ADAR1*^*−/−*^; *ADAR2*^*+/+*^, *ADAR1*^*+/−*^; *ADAR2*^*−/−*^, and *ADAR1*^*−/−*^; *ADAR2*^*−/−*^. Intriguingly, editing activities at these sites decreased dramatically in *ADAR1*^*+/*−^; *ADAR2*^*−/−*^ H1 cells while *ADAR1* ablation leads to complete loss of editing independent of *ADAR2* genotype (Fig. S8). These data strongly point to a dominating role of ADAR1 in mediating A-to-G editing in H1 cells.

### Annotation of RNA editing sites in hESCs

To illustrate the H1 RNA editome, we used ANNOVAR [43] to retrieve annotation for the 11,452 high-quality editing sites (Fig. 2H). Among them, 3761 (33%) are located on intergenic regions, and the rest are on 2600 protein-coding genes and 234 noncoding RNAs (ncRNAs). Six thousand ninety-nine (87%) of the editing sites within protein-coding loci are annotated as intronic, and only two sites are in the coding region of a protein-coding gene *NARF*, but none of them results in nonsynonymous substitution. In contrast, a substantial proportion of ncRNA editing sites are exonic (114 of 706, 16%), which is less appreciated before [44]. After normalization against sequence coverage of RNA sites in each category, it was also found that the editing sites were highly enriched in 3ʹUTR regions compared with other categories (Fig. 2H, *P* < 1.0 × 10^−8^ for every comparison after multiple test correction, Fisher’s exact test). Collectively, these data suggest that RNA editing contributes to sequence diversity of RNA, but not protein, in H1 cells. Three biological processes were enriched in genes carrying the editing sites, including inner cell mass cell proliferation (adjusted *P* < 0.001), blastocyst growth (adjusted *P* < 0.01), and blastocyst development (adjusted *P* < 0.05), consistent with the source of H1 cell derivation. The number of editing sites on each gene also varies substantially. On average, 2.7 editing sites are found per gene. *CYP20A* has the most editing sites (33) and all of these editing sites are in the 3ʹUTR (Fig. S9A). Similarly, nine editing sites exclusively on 3ʹUTR were identified in *LIMD1* (Fig. S9B), a scaffold protein involved in regulation of multiple stem cell signaling pathways including the Hippo pathway and the HIF pathway [45–47].

### Comparative analyses of RNA editomes Upon multilineage differentiation of hESCs

To illustrate the changes of RNA editome upon H1 differentiation, we next retrieved RNA-Seq data of four differentiated cell types derived from H1 from NCBI SRA, including ME, MSC, NPC, and TBL and performed comparative analyses of RNA editomes between H1 cells and four derived lineages. To ensure accuracy in identification of editing events, we used editing sites identified in H1 as query to interrogate RNA-Seq reads in these four cell types, since their corresponding genomic sequences were not available. Qualified sites were defined as those covered by at least five reads. In total, we identified 6757, 4506, 7433, and 5955 A-to-G editing sites in ME, MSC, NPC, and TBL (Supplementary File 2), with a median editing level of 0.21, 0.26, 0.25, and 0.18 separately (Fig. S10A), which is significantly lower than corresponding sites in H1 (0.27, 0.27, 0.29, and 0.27 separately, all *P*-values are <10^–12^, Wilcoxon ranksum test). Notably, the alterations of both the number of editing sites and the median editing levels in the differentiated lineages do not correlate with the expression levels of *ADAR1*, suggesting such distinctions are not simply due to the difference in the abundance of *ADAR1* (Fig. S10B).

One question of particular interest is whether the editing level of the common sites between H1 cells and its derived cells changes upon differentiation. To reduce the random effect between RNA-Seq replicates, we set a stringent criterion that within each cell type, no significant difference in editing level should be observed between replicates using Fisher’s exact test. Then differences between cell types were detected using Poisson test plus a minimum of 2-fold change of the editing level after being normalized against read coverage. As a result, 150 (2.22%), 90 (2%), 459 (6.18%), and 283 (4.75%) sites were found as differentially edited sites (DESs) in ME, MSC, NPC, and TBL respectively (Supplementary File 3).

**Figure 3.**
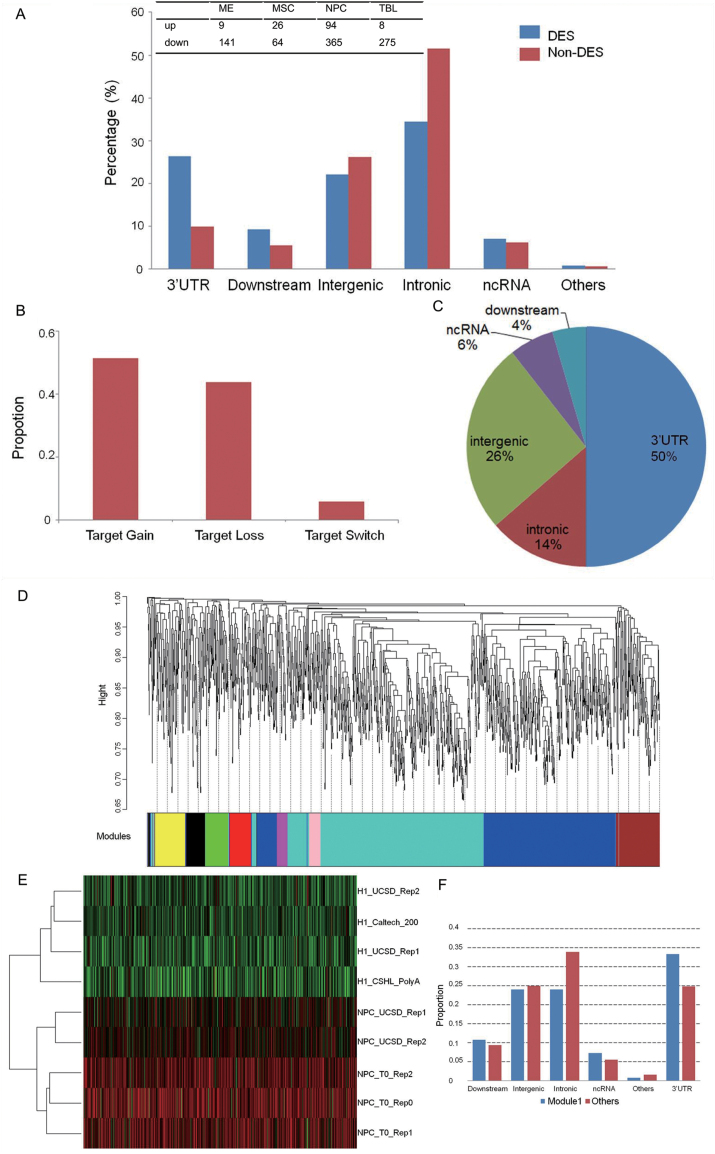
Characterization of differentially edited sites (DESs) and co-editing patterns. (A) The genomic distribution of DESs and nondifferentially edited sites. The inlet table listed the numbers of up- and down-regulated DESs identified in each lineage. (B) The proportion of different types of miRNA target changes for DESs within miRNA target regions. (C) The genomic distribution of DESs in the large sample set. (D) Hierarchical cluster tree of co-edited sites. The color band beneath the tree represents different co-editing modules identified using WGCNA. (E) The heatmap of editing level of module 1 which shows decreased editing in NPC. (F) The functional distribution of editing sites in module 1 compared with other modules.

We further investigated the DESs to obtain better understanding of their potential role in hPSC differentiation. Among them, 141 (94%), 64 (71.11%), 365 (79.52%), and 275 (97.17%) DESs are down-edited in ME, MSC, NPC, and TBL, respectively. DESs are highly enriched in 3ʹUTR regions in these four differentiated lineages (213 out of 810, *P* = 5.4 × 10^−36^, Fisher’s exact test) (Fig. 3A). Considering that miRNAs regulate gene expression by complementing with 3ʹUTR region of mRNAs, it is possible that some RNA editing events in 3ʹUTR may alter miRNA targeting [48]. We thus used miRanda to predict miRNA target sites among them, and found that 171 3ʹUTR DESs were embedded in predicted targets. Among them, potential alterations of targets were profiled (Fig. 3B and Supplementary File 4).

### DESs between H1 and NPC

Due to the importance of RNA editing particularly in the human central neural system, we next focused our comparative analyses on H1 and NPCs and sought to further substantiate our findings by incorporating data from more samples. The same pipeline was applied to two additional H1 samples and three NPC samples with high-quality sequencing data (see Materials and Methods). 1504 editing sites were found across all of the nine samples (Supplementary File 5). Given that these samples are from different experiments, common editing sites across all the samples suggest potential functional importance. Of them, 126 (8.38%) sites were DESs identified above between H1 and NPC in the small sample set (Supplementary File 5), which is significantly higher than what to expect from a random sampling (*P* = 0.002397, Fisher’s exact test).

We next examined whether editing in the set of 126 DESs showed difference between H1 and NPC in the expanded sample set. Sixty-six of 126 (52.4%) DESs were confirmed as showing significant difference in editing level between H1 and NPC (*P* < 0.05, Wilcoxon ranksum test after Benjamini-Hochberg correction), indicating that a substantial number of putative DESs were robustly identified in our pipeline even using a small sample size. We also found that 33 of 66 (50%) DESs identified in the expanded sample set are located in the 3ʹUTR region (Fig. 3C), significantly enriched compared to 117 3ʹUTR sites in 459 NPC DESs using the smaller sample size (*P* = 1.13 × 10^−4^, Fisher’s exact test), which strongly implies RNA editing of 3ʹUTRs may play a major functional role in regulation of NPC differentiation.

### Co-editing network in NPC differentiation

In addition to identification of individual changes, a systematic view of RNA editing would benefit better understanding of its role in NPC differentiation. Therefore, we built a co-editing network using estimated editing level of 1504 editing sites that are found in all H1 and NPC lines. Nearly all sites (1499) were assigned into nine co-editing modules constructed by WGCNA (Fig. 3D). The number of sites within the nine modules range from 32 to 567 (Supplementary File 5). Module 1 contains the most 567 editing sites and shows dramatic down-regulation of editing level in NPC when compared with H1 (Fig. 3E). One hundred eighty-nine sites in module 1 are located in 3ʹUTR, an enrichment only observed in 3ʹUTR in comparison with other regions (0.33 vs 0.25, *P* = 0.0004708, Fisher’s exact test) (Fig. 3F). Those 189 sites pertain to 107 genes, and 51 of them have no 3ʹUTR editing sites in other modules. The identification of such highly correlated editing patterns and cell-specific editing changes within 3ʹUTR regions may infer pivotal editing network in NPC differentiation.

A Case Study of ZYG11B—A Putative Functional RNA Editing Event in NPC Differentiation and its Potential Mechanism of Action.

**Figure 4.**
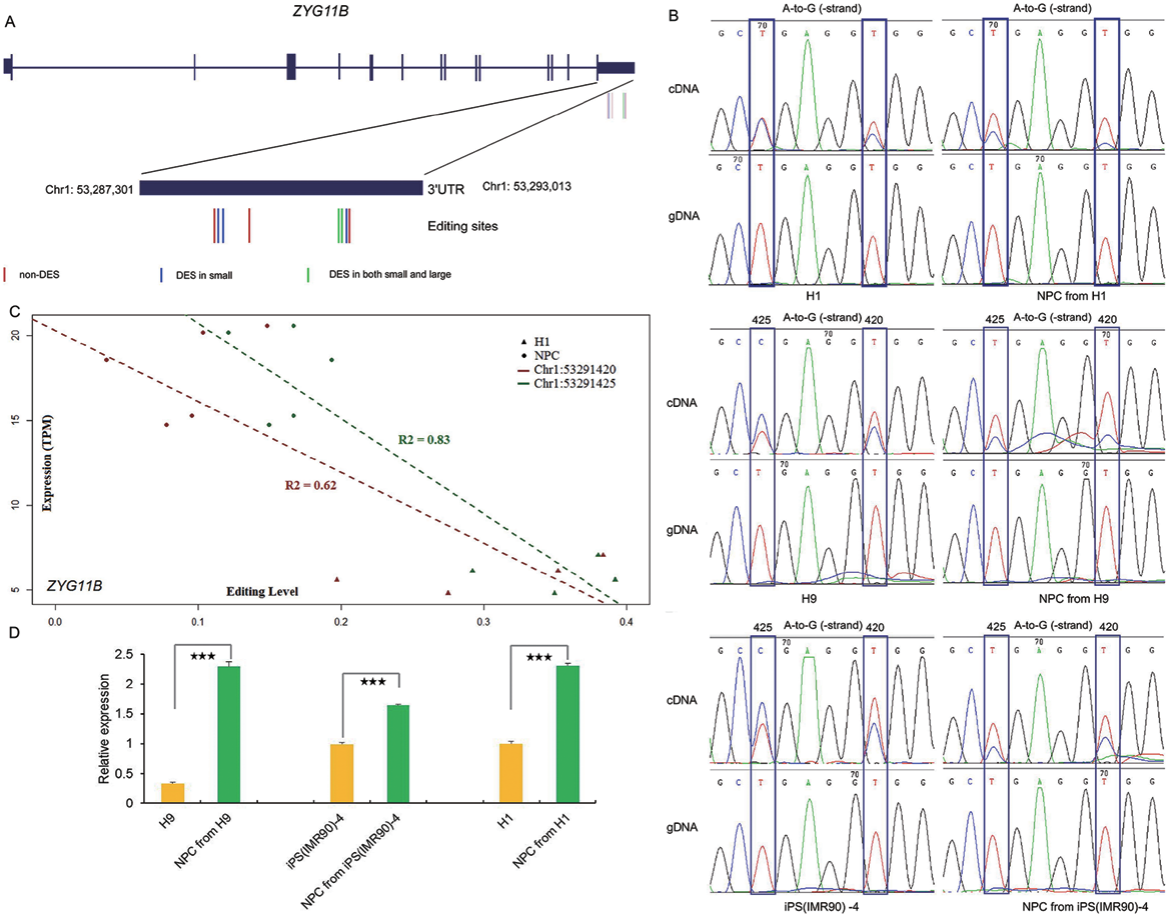
RNA editing sites in ***ZYG11B***. (A) Distribution of RNA editing sites of *ZYG11B* in H1. All eight sites are within 3ʹUTR, three sites are nondifferentially edited sites (red bars), five are differentially edited sites in small sample set (blue and green bars), and two are differentially edited sites in large sample set (green bars). (B) Examination of two DESs from the large sample set (shown with green bars in A) in H1, H9, iPS (IMR90) -4, and derived NPC. The reverse strand is presented. The upper and bottom panels are cDNA and genomic DNA separately. (C) The correlation between the editing levels of two DESs and the expression levels of *ZYG11B* in H1 derived NPC. (D) Relative expression levels of *ZYG11B* in H1, H9, iPS (IMR90) -4, and derived NPC.

Among the genes with robust DESs within 3ʹUTR regions upon NPC differentiation includes *ADRBK2,* which was previously implicated in bipolar disorders and schizophrenia [49, 50]. Furthermore, *ZYG11B* caught our attention for further investigations due to its negatively correlated editing and expression levels in H1 and NPC (Fig. 4). We originally identified eight RNA editing sites in *ZYG11B* using the small sample set, all of which are in the 3ʹUTR region (Fig. 4A). Of them, five are differentially edited between H1 and NPC. In the large sample set, two sites still display significantly different editing frequencies in NPC in comparison to H1, which could be validated by Sanger sequencing (Fig. 4B). Genetic ablation of *ADAR1*, either one or two alleles, leads to loss of editing activity in these two sites, indicating a ADAR1 mediated mechanism (Fig. S8A). Moreover, the editing pattern of these two sites were similar (down-regulated upon NPC differentiation) and negatively correlated with the expression of *ZYG11B* (up-regulated upon NPC differentiation) (Fig. 4C), highlighting their potential function in regulating the mRNA level of *ZYG11B*. These two sites are close in position (chr1:53291420 and chr1: 53291425, latter referred as 420 and 425 sites respectively) but independently edited as only a small portion of sequencing reads that cover both editing sites showed co-editing (Fig. S11). The same negative correlation of editing and expression levels of these two sites were observed in H9 and IMR90-4 hPSC lines and their derivative NPCs (Figs. 4B and 4D), thus implying a conserved regulatory mechanism among distinct hPSC lines.

**Figure 5.**
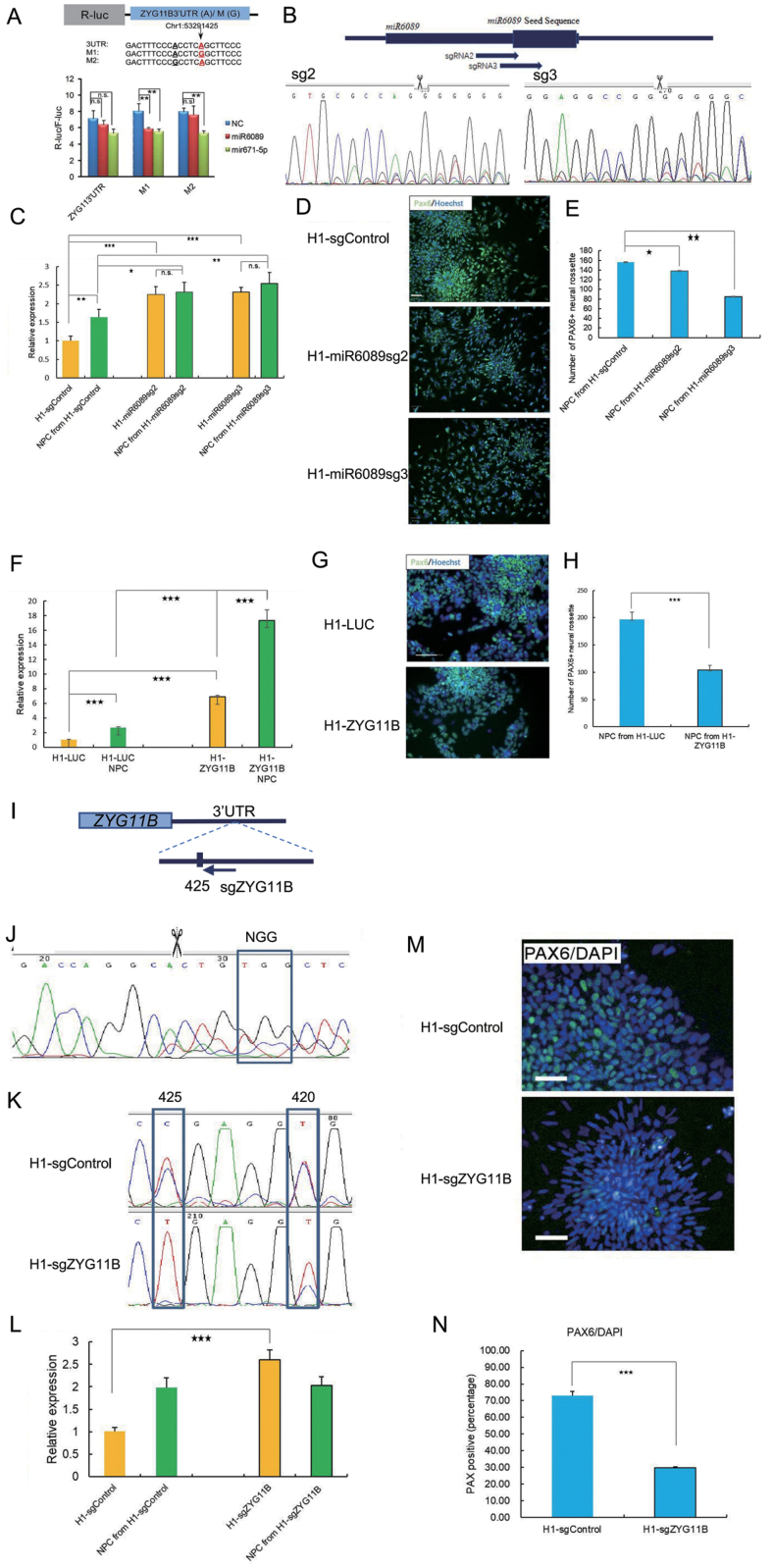
Generation and functional examinations of ***miR6089*** knocking out H1 cell lines and ***ZYG11B*** overexpressing H1 cell line. (A) Cloning of Renilla luciferase reporters fused with part of *ZYG11B* 3ʹUTR containing the two DESs at distinct editing status. The 420 site was highlighted in bold black and the 425 site was highlighted in bold red. Luciferase reporters were cotransfected with plasmids expressing the *miR-6089*, *miR-671-5p* mimics or negative control respectively and Renilla luciferase signal was measured and quantitatively analyzed. (B) Schematic showing *miR6089* seed sequence loci and sgRNA target sites and genome sequencing results. (C) Expression of *ZYG11B* in CRISPR/Cas9 engineered H1 cells and the differentiated NPC cells. (D–E) Representative images of Pax6 (green) and Hoechst (blue) (D) and quantitative analyses (E) of NPCs derived from CRISPR/Cas9 engineered H1 cell lines. Scale bar, 50 µm. (F) Expression of *ZYG11B* in *ZYG11B* overexpressed H1 cell line and the differentiated NPC cells. (G–H) Representative images of Pax6 (green) and Hoechst (blue) (G) and quantitative analyses (H) of NPCs derived from *ZYG11B* overexpressed H1 cell line. (I) Schematic showing *ZYG11B 3ʹUTR 425 editing site* and the adjacent sgRNA target site. (J) Sanger sequencing results of CRISPR mediated genome editing. (K) Examination of the ZYG11B Editing level in CRISPR/Cas9 edited H1 cells. (L) mRNA expression of *ZYG11B* in CRISPR/Cas9 engineered H1 cells and the differentiated NPC cells. Representative images of Pax6 (green) and Hoechst (blue) (M) and quantitative analyses (N) of NPCs derived from CRISPR/Cas9 engineered H1 cell lines. Scale bar, 100 µm. Data are presented as mean ± SD. *n* = 3 Student *t*-test, (*) *P* < 0.05 (**) *P* < 0.01, (***) *P* < 0.001 or not significant (n.s.).

Through miRNA target prediction, we noticed that editing at the 425 site leads to potential target gain of *miR-6089* and *miR-671-5p*. We then examined the expression of *miR-6089* and *miR-671-5p* in H1 cells and their derivative NPCs. The qPCR results showed that both *miR-6089* and *miR-671-5p* are expressed in H1 and NPCs. And *miR-6089* is significantly more abundant in both cell types (Fig. S12). To examine whether any of these two miRNAs would differentially target the *ZYG11B* 3ʹUTR in distinct editing status, we cloned a series of luciferase reporters fused with a 522 bp portion of the 3ʹUTR region of *ZYG11B*, in which the 420 and 425 sites are either A or G. Using these reporters, we observed that a G in the 425 site indeed led to gain of *miR-6089* mediated repression, while *miR-671-5p* mediated knockdown appeared not dependent on the editing status (Fig. 5A).

To further investigate potential role of *miR-6089* in regulation of *ZYG11B* and NPC differentiation, we designed two sgRNAs targeting the seed sequence of *miR-6089* (Fig. 5B). Stable polyclonal H1 cell lines were generated upon lentiviral infection with particles harboring Cas9 and each sgRNA. Sanger sequencing validated indels in the seed region derived from NHEJ mediated by CRISPR/Cas9 (data not shown). *ZYG11B* mRNA level was significantly elevated in both H1 cell lines delivered with *miR6089* disrupting sgRNAs and their NPC derivatives in comparison with cells delivered with a unrelated control sgRNA (Fig. 5C), a finding consistent with *miR6089* targeting of edited *ZYG11B* mRNA. NPC differentiation of both *miR6089* disrupted H1 cell lines was also significantly impaired, with the sgRNA3 being more effective (Figs. 5D and 5E), consistent with its target site closer to the core of *miR6089* seed sequence (Fig. 5B). To further validate whether the impairment of NPC differentiation upon *miR6089* perturbation is due to overexpression of *ZYG11B*, we generated a stable *ZYG11B* overexpressing H1 cell line (Fig. 5F) and found that its NPC differentiation was indeed affected in comparison with a luciferase expressing control (Figs. 5G and 5H). To precisely disrupt the editing event at site 425, we used a sgRNA that targets its adjacent sequence and successfully abolished 425 editing (Figs. 5I–K). Consistent with the site as a microRNA target, ZYG11B expression was up-regulated (Fig. 5L) and further, its NPC differentiation efficiency was impaired (Figs. 5M and 5N).

**Figure 6.**
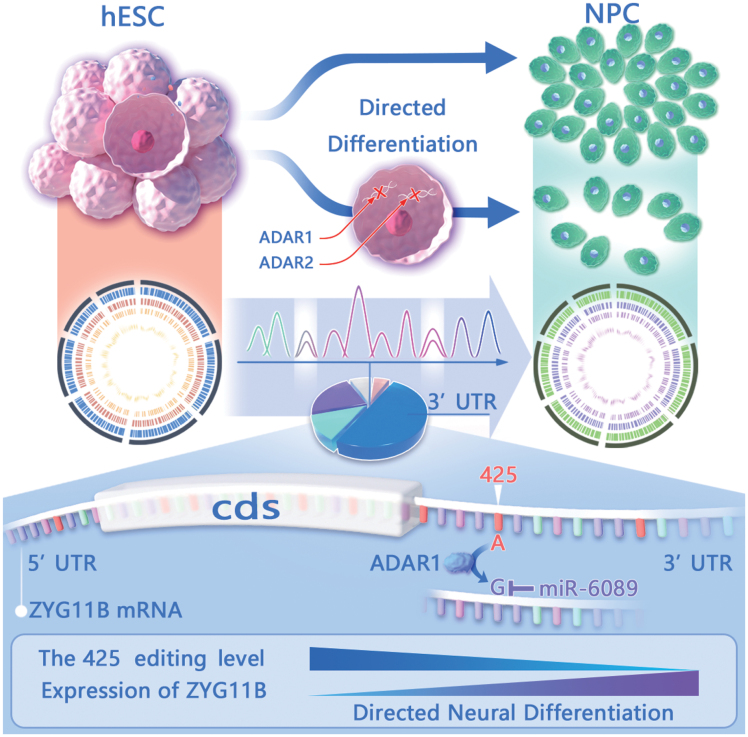
Graphical summary. Genetic knockout of RNA editing enzymes ADAR1 and 2 in hESC leads to impaired NPC differentiation, an indication of functional importance. Comparative editomes were profiled and a potential regulatory RNA editing event was functionally characterized. *MiR6089* specifically targets the edited 425 site on *ZYG11B* 3ʹUTR, conferring a reverse correlation of its editing level and *ZYG11B* expression level, regulation of which might play a role in modulating NPC differentiation.

Collectively, guided by bioinformatics analyses, we identified a rewiring event of *miR-6089* targeting derived from differential editing in the 425 site at the 3ʹUTR of *ZYG11B* (Fig. 6). This provided a plausible explanation of the negative correlation between the editing and expression levels of *ZYG11B* upon NPC differentiation. Furthermore, we provided evidences for a regulatory function of *miR6089* through acting on *ZYG11B* mRNA in NPC differentiation (Fig. 5).

## Discussion

In the current study, we presented evidences for important regulatory functions of A-to-G RNA editing in NPC differentiation through *ADAR* knockout using a CRISPR/Cas9 approach (Figs. 1, S1 and S5). We then profiled the A-to-G editome in H1 cell line (Figs. 2 and S6–S8), and then conducted a series of comparative analyses between the RNA editomes in H1 cells and their multilineage directed differentiation (Figs. 3, S9 and S10). We then focused on differentiation to the neural fate and further substantiated our pattern analyses by recruiting RNA-Seq data from additional samples (Fig. 3). Guided by multi-dimensional analyses of DESs, miRNA targeting predictions of editing sites on 3ʹUTRs, and RNA-editing network (Fig. 3), we further focused on a case study of the 425 editing site on the 3ʹUTR of *ZYG11B* (Figs. 4 and S11). Consequently, an editing derived *miR6089* rewiring event at the 425 site was identified, for which the functional importance of *miR6089* and *ZYG11B* in regulating NPC differentiation was also implicated by targeted disruption of the seed region of the miRNA and overexpression of *ZYG11B* (Figs. 5 and S1^2^).

Interestingly, only two out of over 10 thousand editing sites were located in protein-coding region from our analysis on H1, consistent with a previous report of enriched m6A modification in coding regions leading to decreased association with ADAR enzymes [41]. Furthermore, none of these two editing events leads to amino acid substitution, in contrast to frequent nonsynonymous alterations reported in multiple pathological conditions of neurodegenerative diseases and cancers [32, 51–56]. It is then intriguing to speculate that coordinated and tight regulation of two most abundant RNA modifications both on adenosine, A-to-G editing and m6A, in protein-coding regions, might play a role in maintaining a nonpathological state.

In contrast to very rare editing events in coding regions, multi-dimensional analyses in the current study identified enrichment of A-to-G editing activity in 3ʹUTR. Furthermore, guided by these analyses, we identified a potential functional editing event in the 425 site of *ZYG11B* 3ʹUTR that leads to gain of *miR6089* knockdown (Fig. 5). Consistent with our findings, other cases of editing derived alterations of miRNA targeting have been reported previously [39, 57].

Above all, better understanding of RNA editing events and their change during human neural differentiation might shed new lights in human neural development and multiple neurological and psychiatric disorders, including schizophrenia, bipolar disease, autism, epilepsy, amyotrophic lateral sclerosis, and depression [21–30]. Considering the rapid development of targeted RNA editing tools such as CRISPR-Cas13 and the LEAPER system, investigations of endogenous RNA editing events might identify potential opportunities for gene therapy, for which intervention at the RNA level is reversible, thus considered to be safer in comparison to permanent editing of genomic DNA [58, 59].

## Research limitations

hPSC differentiation in vitro might not accurately reflect the embryonic development *in vivo*. Therefore, correlation of the findings from this study with human neural development *in vivo* is still being defined. In addition, we relied on CRISPR/Cas9 based genetics for functional perturbations, targeted modulation of RNA editing activity with the latest CRISPR-Cas13 and LEAPER system would deliver more mechanistic insights [58, 59]. Moreover, we only focused on ZYG11B editing events in the current study. Further studies on additional events profiled here (Supplementary File 5) and their underlying mechanisms would provide more insights of the function of RNA editing in NPC differentiation.

## Materials and methods

### Generation of CRISPR/Cas9 engineered H1 cell lines

Two sgRNAs targeting the coding region of *ADAR1*, two for *ADAR2*, and two targeting miR6089 seed sequence were designed and cloned respectively into lentiviral expressing plasmids harboring a blasticidin selection cassette [60]. These sgRNA sequences were listed in Supplementary File 1. A lentiviral expression plasmid containing human codon optimized *Streptococcus pyogenes* Cas9 tagged with nucleus localization signals, which co-expresses puromycin resistant gene [60] was used in combination with sgRNAs for *genome editing*.

Lentivirus particles were packaged in HEK293T cells by co-transfection with psPAX2 and PMD2.G plasmids and introduced to H1 cell culture. Six micrograms per milliliter polybrene (Cat# H9268, Sigma) was applied to enhance infection efficiency. Meanwhile, 10 μM Y-27632 (Cat# S1049, Selleck) was added to the medium to enhance single cell survival. Upon antibiotic selection using 2 μg/mL puromycin (Cat# A1113802, Life Technologies) and 1 μg/mL blasticidin (Cat# R21001, Life Technologies), genome edited H1 cells were enriched. Monoclonal cell lines were picked and analyzed for *ADAR* knockout. Polyclonal H1 cell line was obtained and analyzed for *miR6089* knockout. A control sgRNA with an unrelated targeting sequence was used as a negative control.

### Generation of *ZYG11B* overexpressing H1 cell line

The human *ZYG11B* CDS was PCR-amplified from H1 cDNA and cloned into a lentiviral expression plasmid harboring puromycin selection marker. A luciferase expressing control plasmid was generated in parallel. Lentivirus particles containing *ZYG11B* or luciferase expression cassette were produced and introduced to H1 cell culture. Six micrograms per milliliter polybrene (Cat# H9268, Sigma) was used to enhance infection efficiency. Meanwhile, 10 μM Y-27632 (Cat# S1049, Selleck) was added to the medium to enhance single cell survival. Upon antibiotic selection using 2 μg/mL puromycin (Cat# A1113802, Life Technologies), polyclonal H1 cell lines was obtained.

### hPSC culture and differentiation

hESC line H1 and H9 and hiPSC line IMR90-4 were obtained from WiCell and cultured in the chemically defined E8 medium following manufactor’s manual (Life Technologies). The differentiation of ME, TBL, and NPC were conducted following the protocols from Xie et al. [35]. H1 cells were passaged by EDTA (Cat# 25200072, Life Technologies), 2 days before differentiation. As for ME differentiation, H1 cells were passaged using TrypLE (Cat# 12604021, Life Technologies) onto plates coated with Matrigel (Cat# 354277, BD Biosciences) and cultured in E8 with 5 ng/ml BMP4 (Cat# 120-05ET, Peprotech), and 25 ng/mL Activin A (Cat# 120-14P, Peprotech). Cells were harvested at the end of day 2. For differentiation toward TBL lineage, H1 cells were passaged by EDTA and cultured in E8 without FGF2 and with 50 ng/mL BMP4 added to the medium. Differentiated cells were collected at the end of day 5. For NPC differentiation, H1 cells were passaged by EDTA and cultured in E8 without FGF2 and TGFβ1, but with 10 µM SB431542 (Cat# S1067, Selleck) and 100 ng/mL Noggin (Cat# 14772-1-AP, Proteintech) added to the medium. Differentiated cells were harvested at the end of day 7.

### Immunofluorescence

Cells were fixed in 4% paraformaldehyde for 20 min and permeabilized in 0.3% Triton X-100 for 15 min and then blocked with 2% bovine serum albumin (Cat# A6003, Sigma) for 1h. Samples were subsequently incubated with primary antibodies overnight at 4°C, and then secondary antibody for 1h at room temperature. Finally nuclei were stained with Hoechst 33342 (Cat# H1399, Thermo Fisher). The images were collected on operetta high content scanner (Perkin Elmer). Primary antibodies used include anti-Oct4 (1: 200, Cat# SC5279, Santa Cruz), anti-Nanog (1: 200, Cat# ab80892, Abcam), anti-Sox2 (1: 200, Cat# 651901, Biolegend), and anti-Pax6 (1: 1000, Cat# 901301, Biolegend).

### Genomic and transcriptomic data

SOLiD whole genome sequencing reads (50 bp) for the H1 cell line and Illumina HiSeq2000 transcriptomic sequencing reads (101 bp) for H1 and its derivatives including ME, MSC, NPC, and TBL generated in UCSD Human Reference Epigenome Mapping Project [35] were downloaded from NCBI short read archive database (http://www.ncbi.nlm.nih.gov/sra). All the access numbers can be found in Supplementary File 1. As previously reported [35], RNA was isolated from H1and differentiated cells including ME, MSC, NPC, and TBL using Trizol (Life technology). The deep sequencing libraries were constructed using the TruSeq RNA Sample Prep Kit (Cat# RS-122-2002, Illumina).

We also analyzed additional RNA-Seq datasets generated by the same protocol to increase statistical power of comparison. For H1 cell line, we included two ENCODE datasets GSM958733 and GSM758566. We also examined two additional H1 datasets (GSM958733 and GSM758573), but only a small proportion of editing sites were covered due to limited sequencing depth, we therefore decided not to use them to minimize information loss. For NPC cell line, we used three biological replicates from H1-derived NPC [61]. Multiple runs for one library were merged together. Since various experimental factors could affect the RNA-Seq process, we also performed clustering analysis based on gene expression estimated by RSEM (Fig. S13), and confirmed that all samples used in this study were clustered by cell type, thus few bias was expected by compiling different datasets.

### Identification of RNA editing sites

We first identify RNA editing sites in UCSD H1samples using a modified pipeline developed in previous studies [38, 40]. Briefly, Burrows-Wheeler Aligner (BWA) version 0.5.9 [62] was used to align both Illumina reads and color-spaced SOLiD reads. To align Illumina RNA-seq reads, we combined as the reference the human genome reference (hg19) and 200-bp exonic sequences flanking known splicing junctions (1-bp shorter than the Illumina reads on each side) for Gencode, RefSeq, Ensembl, and UCSC annotated genes, and mapped each of the paired-end reads separately by setting the maximum edit distance in the seed as 1 (*bwa aln -k 1*). For color-space SOLiD reads, we aligned them against hg19 reference genome directly using the command “*bwa aln -c -k 1.*” Only uniquely mapped reads with a mapping quality ≥ 20 and ≤ 5 mismatches were considered and potential PCR duplicates were removed by SAMtools [63].

We next used “*samtools mpileup*” to recalculate base alignment quality (BAQ) and identified single nucleotide variants supported by at least five RNA-Seq reads including two variant reads with a BAQ ≥ 25 and a mapping quality ≥ 20. We also set the minimum read cutoff as 10 or 20, but similar pattern was observed thus in this study, we only present the results using a cutoff of 5. Filters were applied to remove variants that were (i) found in the genomic reads; (ii) at the first six bases in RNA-seq reads (false positives could be introduced by random-hexamer priming in Illumina reads); (iii) known SNPs in dbSNP database (http://www.ncbi.nlm.nih.gov/SNP/, version 137), 1000 Genomes Project (https://www.internationalgenome.org), or the University of Washington Exome Sequencing Project (http://evs.gs.washington.edu/EVS/). Similar with the previous study [40], we observed an extremely high A-to-G fraction for variants in *Alu* regions, and only applied additional filters for non-*Alu* regions, including removing (i) variants with low frequency (<0.1); (ii) intronic variants within 4 bp of known splicing junctions; (iii) variants in homopolymer runs of ≥ 5 bp; and (iv) variants in regions highly similar to other part of the genome. All these additional filters are the same as the previous study and details can be found therein [40].

To obtain the directionality of each RNA editing site, we examined all transcripts overlaying the editing site and the strand information was assigned only when all transcripts were on the same strand. ANNOVAR [43] was used to annotate each editing site.

Since the genomic sequences for other samples were not available, we then used editing sites identified above as covered by at least query to interrogate RNA-Seq reads in those samples and defined editing sites as ones five reads with a mapping quality ≥ 20 and a base quality BAQ ≥ 25.

### Validation of potential RNA editing sites by Sanger sequencing

Sanger sequencing was used to validate the candidate editing sites in hPSC cell lines, including H1, H9, and IMR90-4, and derived NPCs. We obtained total RNA and genomic DNA using Trizol kit (Cat# 15596026, Invitrogen) and genomic DNA isolation kit (Cat# A2360, Promega), respectively. One microgram total RNA was used for reverse transcription following the instruction of reverse transcription system (Cat# M1705, Promega). Typically a 25 μL PCR reaction was assembled using ~10 ng genomic DNA or ~5 ng cDNA as template, 10 μM forward and reverse primers, and 0.5 U KOD (Cat# KOD-401, TOYOBO) polymerase. The following PCR program was used to amplify the target: 94°C for 2 min; 30 cycles of 94°C for 15 s, 60°C for 30 s, and 68°C for 1 min; and finally 68°C for 10 min. PCR products were purified from 1% agarose gel, and sent for Sanger sequencing (The Beijing Genomics Institute) quantitively analyzed by LightCycler 480 II (Roche). The PCR primers were listed in Supplementary Files 3–5.

### Prediction of miRNA targets

We used miRanda algorithm (release August 2010) to predict miRNA targets. Similar to a previous study [64], we extracted 30-bp up- and down-stream sequences for each editing position and constructed two 61-bp query sequences using both the reference allele and the editing allele to feed into the miRanda program. The alignment score threshold was set to 155 and the energy threshold was set to −20, all the other parameters were set as default. The 2578 human mature miRNA sequences were retrieved from miRBase (www.mirbase.org/, release 20). A predicted target was retained if it covered the editing position.

### Co-editing network analysis

WGCNA software [65] was used to perform weighted co-editing network analysis [66]. Briefly, pairwise correlations were first calculated between all possible pair of editing sites across all samples, and then were used to construct the adjacency matrix using a soft-threshold value of 12. Next topological overlap matrix was constructed using the above adjacency matrix, and co-editing sites were clustered together by average linkage hierarchical clustering. The resulting clusters were cut by the dynamic hybrid tree cut algorithm and branches under the cut were defined as different modules [67]. For each module, a module eigen-value was derived as the first principle component. Highly correlated modules (in terms of module eigen value) were further merged using mergeCutHeight of 0.25.

We next identified intramodular hub editing sites by calculating module eigen value-based connectivity or module membership (MM). For a given editing site *i*, MM^*q*^(*i*) = cor(x(*i*), *E*^*q*^), where *q* is the module, x(*i*) denotes the expression level, and *E*^*q*^ is the module eigen value for module *q* [68, 69]. Hub editing sites were defined as those with highest MM values (MM > 0.9 for a module).

### *ZYG11B* 3ʹUTR luciferase assay

*ZYG11B* 3ʹUTR sequences containing the 420 and 425 sites were PCR-amplified from human ES H1 cDNA using forward primer: CCGCTCGAGGGTTGAGGTGAATAAAGCTGCAT and reverse primer: ATAAGAATGCGGCCGCCCTTGGGTGCAGCTGTAC, which was designed to include XhoI and NotI restriction sites. The PCR products were cloned into the psiCHECK-2 [70, 71] dual-luciferase vector using XhoI/NotI sites. Clones that displaying three distinct editing status shown in Fig. 3 were obtained simultaneously. The obtained constructs were cotransfected with *miR671_5p* and *miR6089* mimics into NPC cells separately using Fugene (Cat# E2311, Promega). Luciferase expression was detected using the dual-luciferase reporter system (Cat# E2920, Promega) according to manufacturer’s protocol. Renilla luciferase activity was normalized against firefly luciferase activity, an internal control for transfection efficiencies.

### RNA isolation, RT-PCR, and qPCR

Total RNA was isolated and reversed transcribed using Trizol kit (Cat# 15596026, Invitrogen) and reverse transcription system (Promega) following the manufacturer’s protocols. SYBR Green (Cat# QPK-201, TOYOBO) was used for qPCR analyses. Hairpin-it^TM^ microRNA and U6 snRNA Normalization RT-PCR Quantitation Kit (Cat# E01011, GenePharma) was used to examine miRNA expression. Primers were listed in Supplementary File 2.

### Statistical analysis

Unless otherwise specified, all statistical analyses were performed by R [72].

### Data availability

All data included in this study are available upon request by contact with the corresponding author.

## Supplementary Material

lnac027_suppl_Supplementary_Material

lnac027_suppl_Supplementary_File_1

lnac027_suppl_Supplementary_File_2

lnac027_suppl_Supplementary_File_3

lnac027_suppl_Supplementary_File_4

lnac027_suppl_Supplementary_File_5
