# Supplementary material for: Comparative functional RNA editomes of neural differentiation from human PSCs
Source: Life Med. 2022 Aug 10;1(2):221–35. doi: 10.1093/lifemedi/lnac027 (PMC11749364; doi:10.1093/lifemedi/lnac027)
Supplement: lnac027_suppl_Supplementary_Material [file lnac027_suppl_Supplementary_Material.docx]

**Comparative Functional RNA Editomes of Neural Differentiation from Human PSCs**

**Yu Zhang^1,4,^**^#^**, Qu Zhang^1,5,^**^#^**^,^*, Yuhong Hou^2,3,6,^**^#^**, Ran Wang^2,3,7^, Yu Wang^1,^***

*^1^College of Life Sciences and Oceanography, Shenzhen University, Shenzhen 518055, China*

*^2^State Key Laboratory of Stem Cell and Reproductive Biology, Institute of Zoology, Chinese Academy of Sciences, Beijing 100101, China*

*^3^University of Chinese Academy of Sciences, Beijing 100049, China*

*^4^Mlobio, Singularity Center，Beijing 102200，China*

*^5^Experimental Medicine Unit, GlaxoSmithKline, Collegeville, PA 19426, USA*

*^6^Cell Resource Center, Peking Union Medical College (PUMC), Beijing 100005, China*

*^7^Peking Union Medical College Hospital, Beijing 100730, China*

^#^*These authors contributed equally to this work.*

*Corresponding authors. E-mail: yu-wang@szu.edu.cn (Y.W.) and E-mail: quzhang@post.harvard.edu (Q.Z.)

**Supplemental Information**


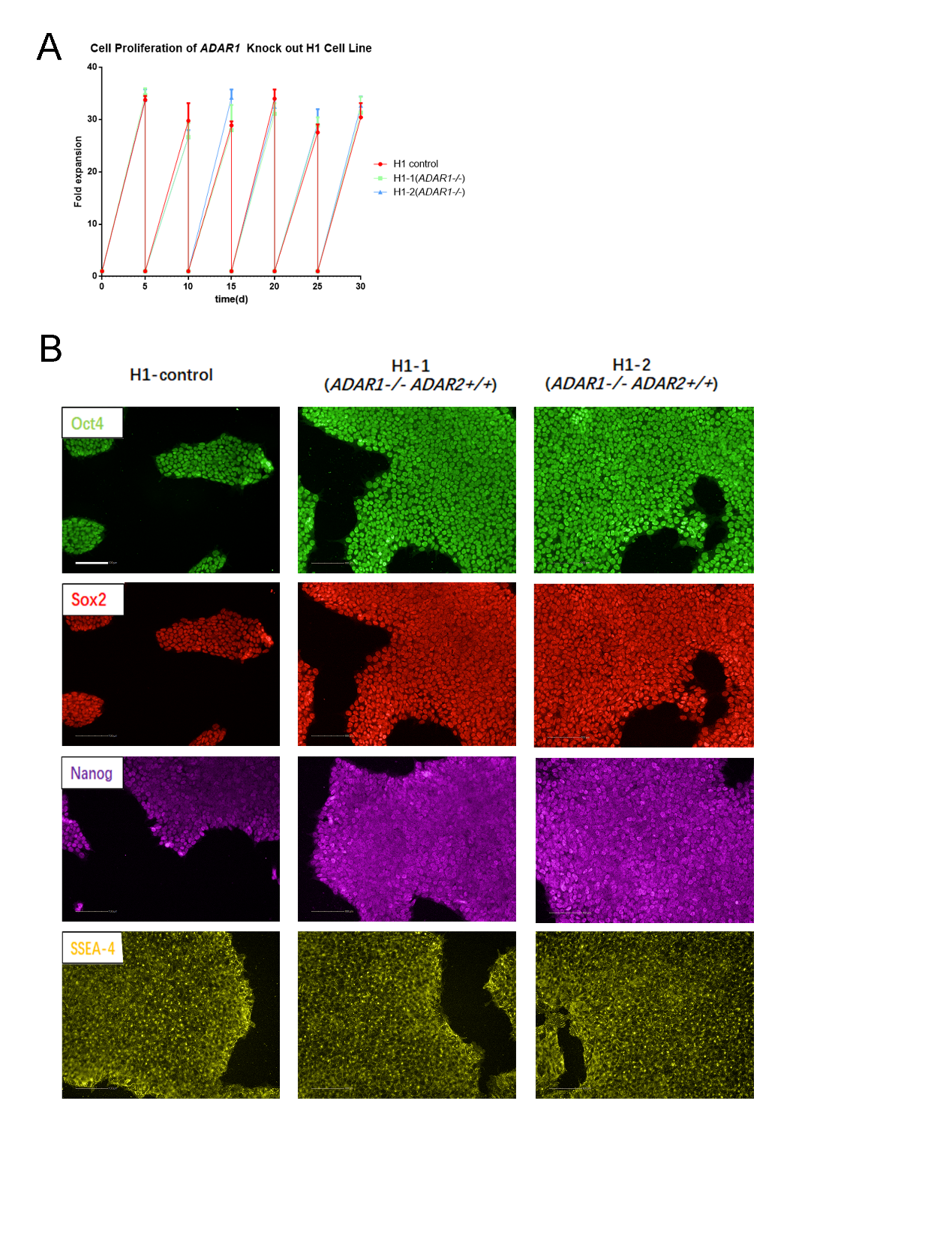


**Supplemental Figure 1. Characterization of *ADAR1* knock out H1 cell lines.**

(A) Fold of expansion of ADAR1 knock out H1 cells and control cells. 30000 cells were plated at each passage. (B) Immunofluorescence of pluripotency markers SOX2, OCT4, Nanog and SSEA-4 in ADAR1 knock out H1 cells. Scale bar, 100 μm. Data are presented as mean ± SD. *n* = 3.

**
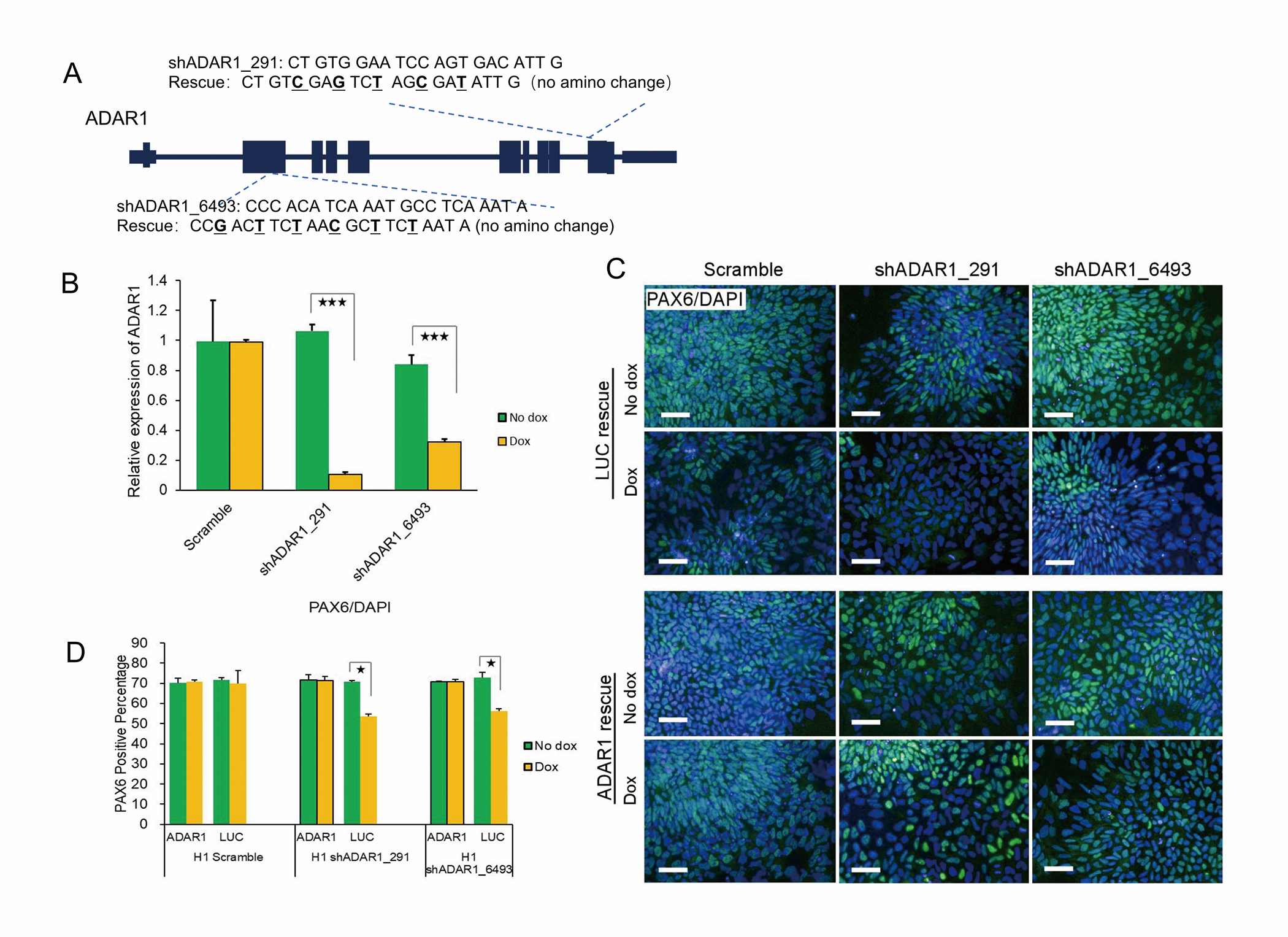
**

**Supplemental Figure 2. ADAR1 knockdown and rescue.** (A) Schematics showing *ADAR1* loci and 2 shRNA target sites, and the recoding sequence for rescue. (B) Examination of ADAR1 mRNA level upon shRNA knockdown. (C) Representative images of Pax6 (green) and Hoechst (blue) and quantitative analyses (D) of NPC differentiation upon ADAR1 knockdown and rescue using recoded sequence shown in (A). Data are presented as mean ± SD. *n* = 3 Student *t*-test, (*) *P* < 0.05.

*
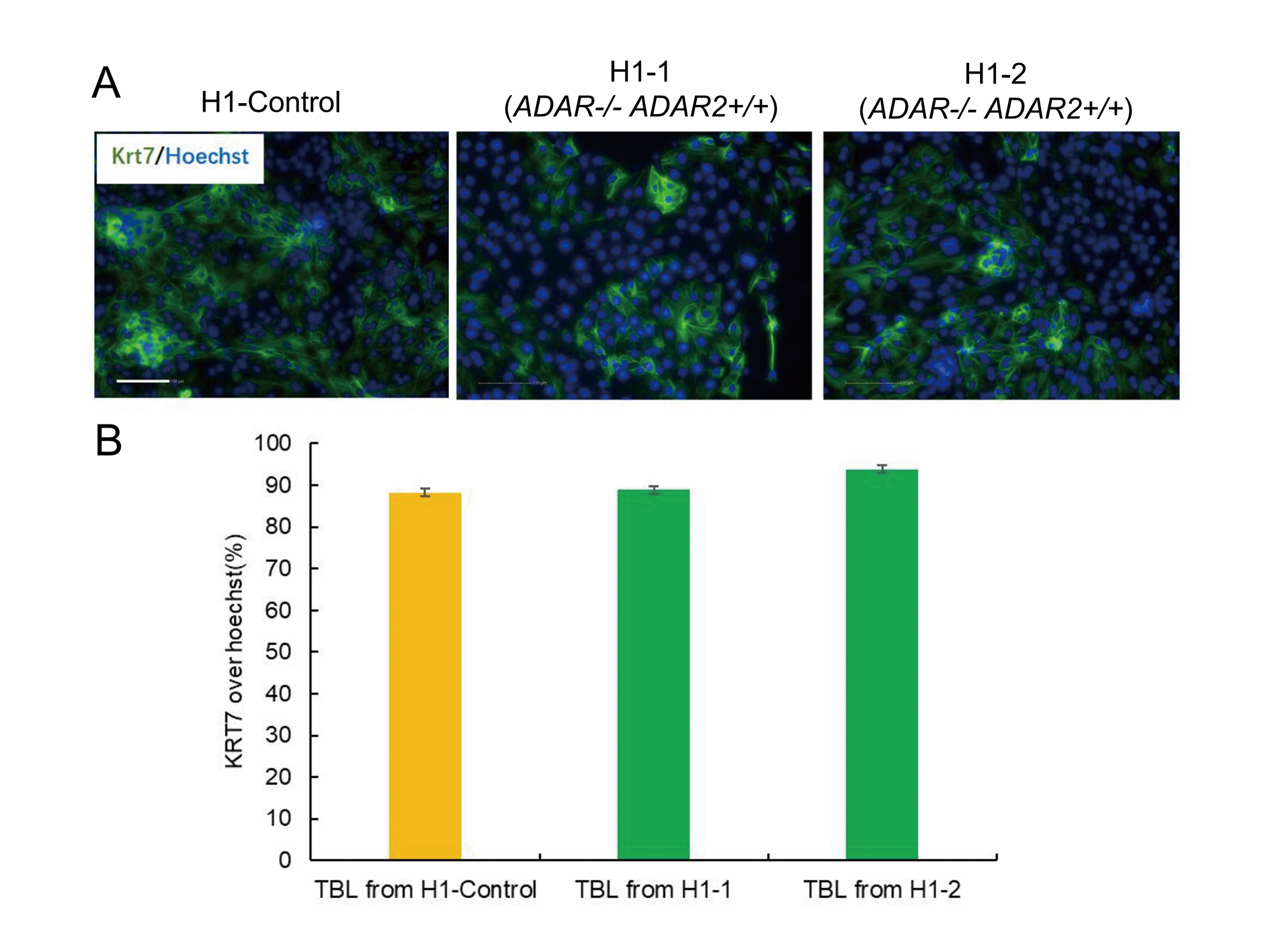
*

**Supplemental Figure 3. TBL differentiation of *ADAR1* knock out H1 cell lines.**

(A-B) Representative images (A) and quantitative analyses (B) of TBLs derived from two ADAR1-/- monoclonal H1 cell lines (H1-1 and H1-2), in comparison with a wildtype H1 cell line (H1-control) generated from the same round of CRISPR/Cas9 genome editing. Krt7 (green) was used as a specific marker for TBL. Hoechst (blue) was used to stain the nucleus. Scale bar, 100 µm. Data are presented as mean ± SD. *n* = 3.


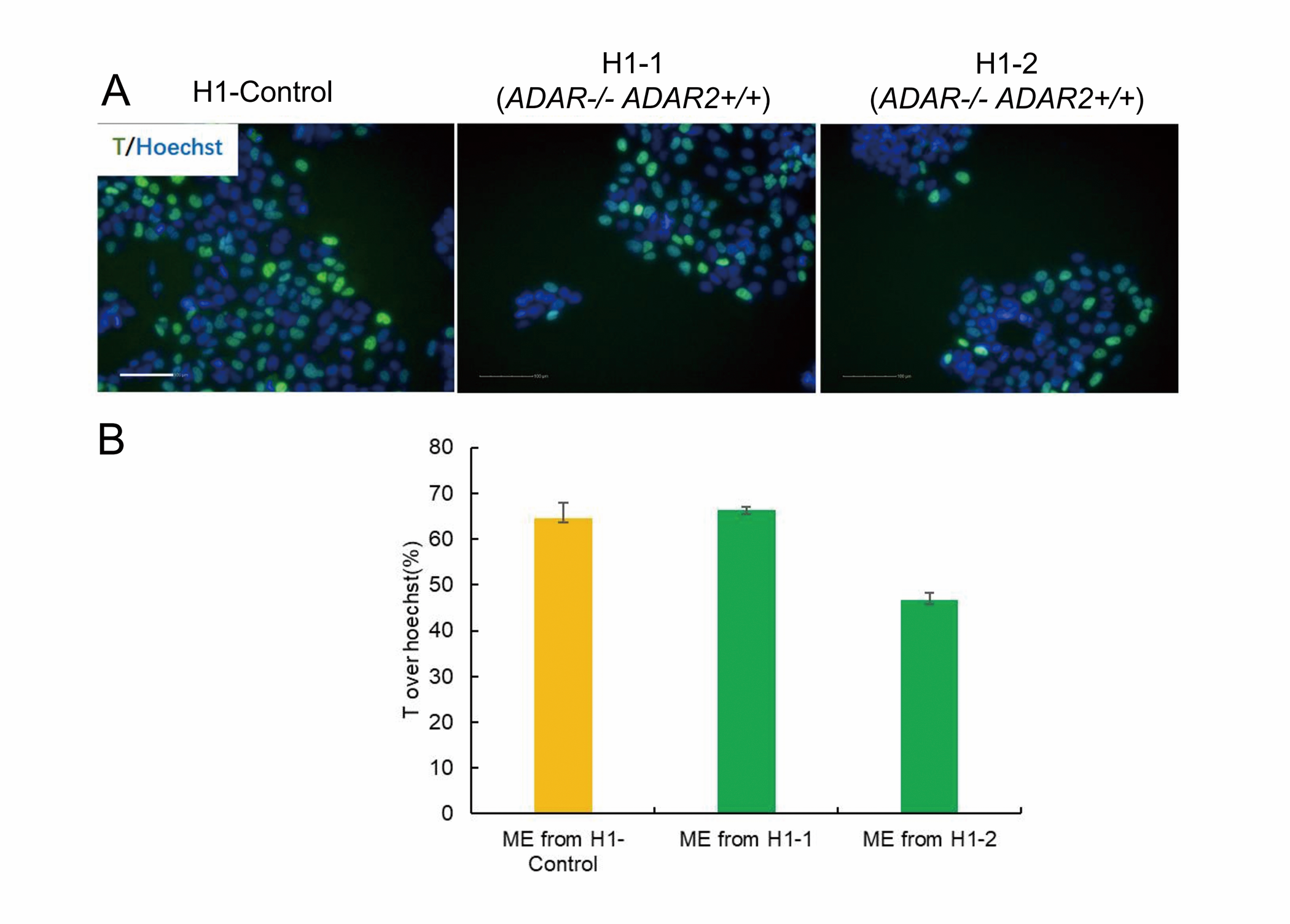
**Supplemental Figure 4.** **ME differentiation of *ADAR1* knock out H1 cell lines.**

(A-B) Representative images of T (BRACHYURY, green, specific marker of ME) and Hoechst (blue) (A) and quantitative analyses (B) of ME derived from two ADAR1-/- monoclonal H1 cell lines (H1-1 and H1-2), in comparison with a wildtype H1 cell line (H1-control) generated from the same round of CRISPR/Cas9 genome editing. Scale bar, 100 µm. Data are presented as mean ± SD. *n* = 3.

**
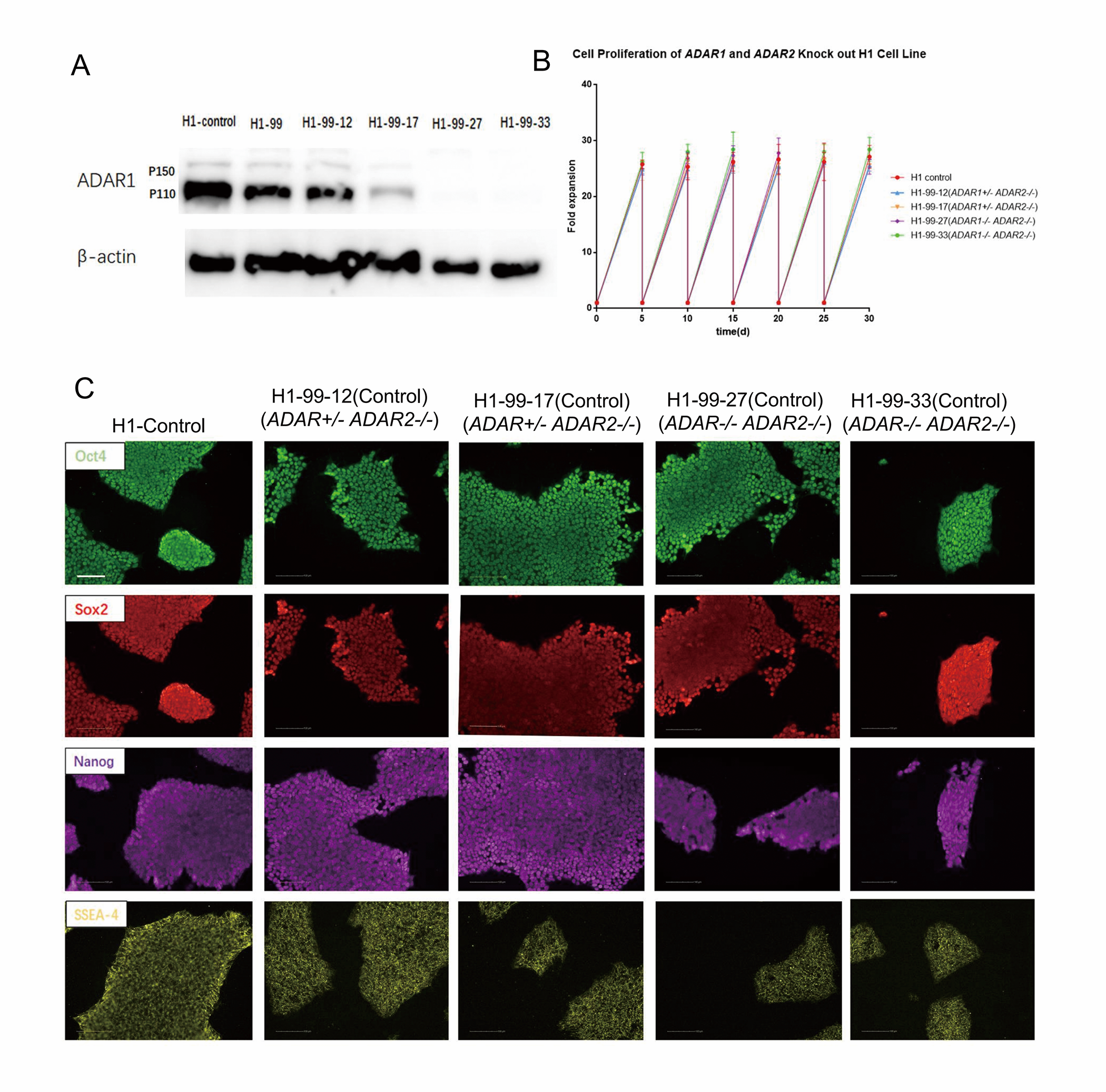
**

**Supplemental Figure 5. Characterization of *ADAR1/2* compound knock out H1 cell lines.**

(A) Western blotting (WB) confirmed *ADAR1/2* genotypes in H1 cell lines. Actin was used as a loading control. (B) Fold of expansion of *ADAR1/2* knock out H1 cells and control cells. 30000 cells were plated at each passage. (C)Immunofluorescence of pluripotency markers SOX2, OCT4, Nanog and SSEA-4 in *ADAR1/2* knock out H1 cells. Scale bar, 100μm. Data are presented as mean ± SD. *n* = 3.


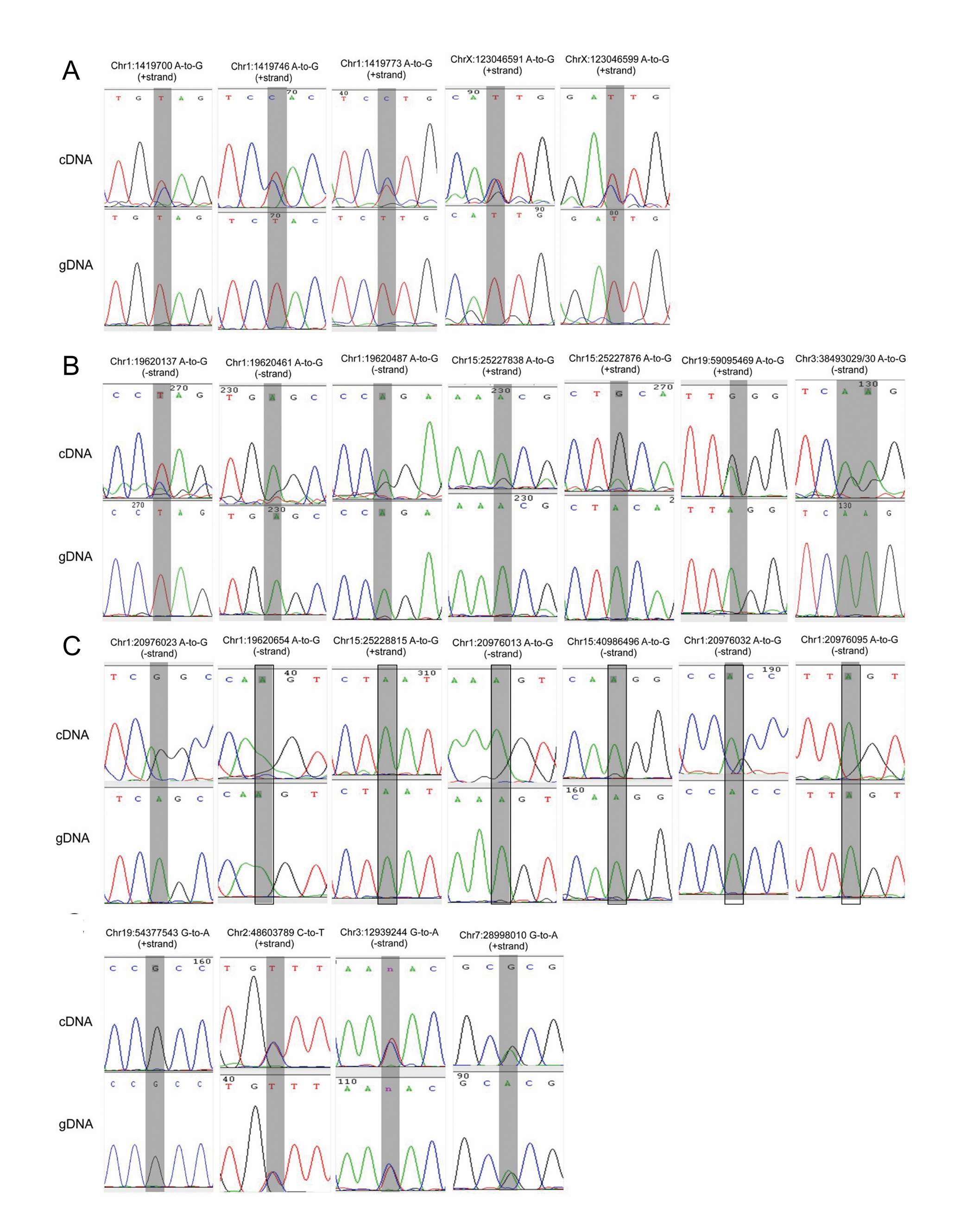


**Supplemental Figure 6. Validation of editing sites by Sanger sequencing in H1 cells.**

(A) Validation of the editing sites in coding genes by Sanger sequencing. (B) Validation of the editing sites in lncRNA by Sanger sequencing. (C) Examination of inferred non-canonical editing sites by Sanger sequencing. The sites were highlighted by gray shading. False positive editing was indicated by square frame with gray filling. Top and bottom panels are results from cDNA and genomic DNA respectively.


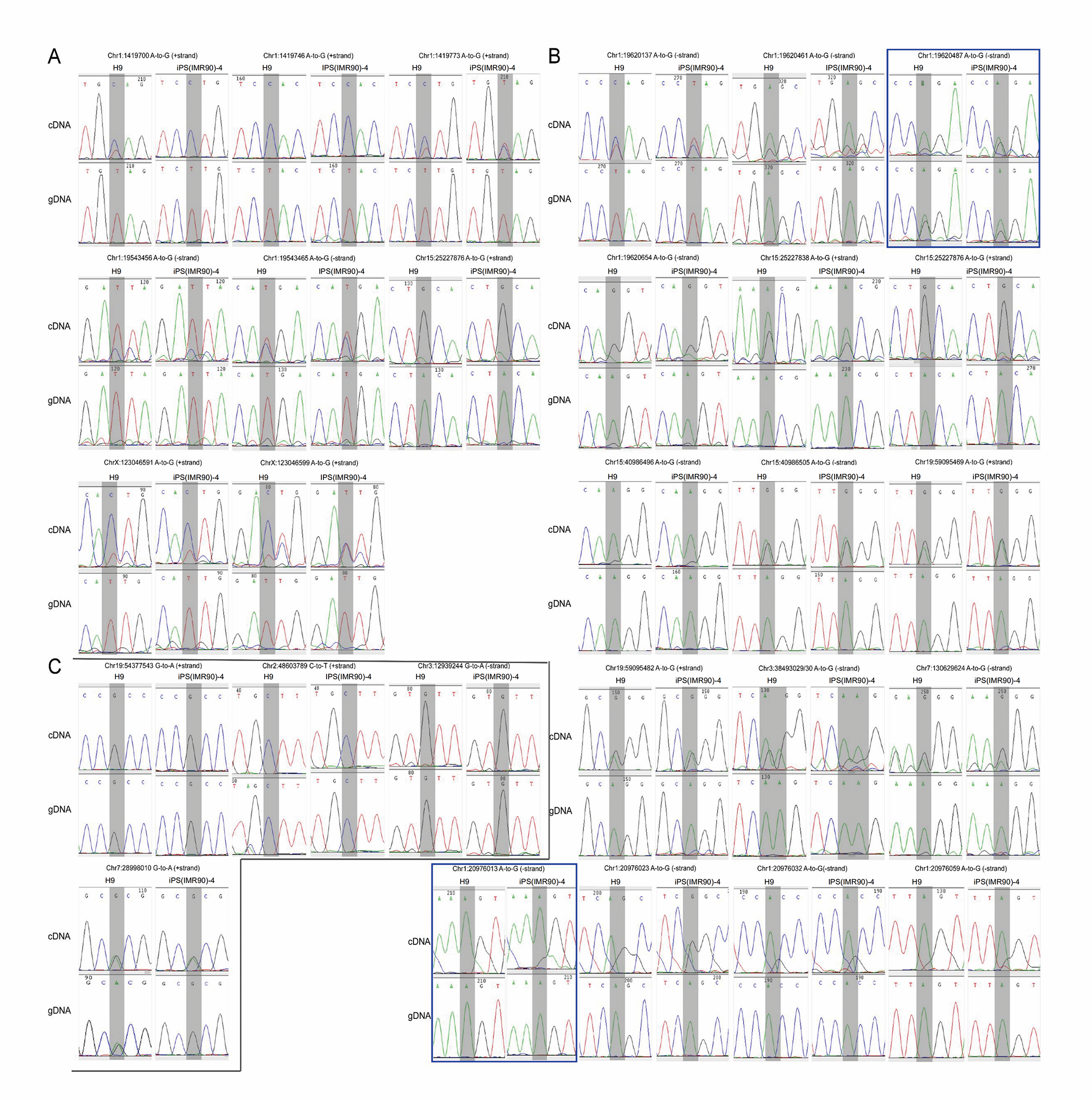


**Supplemental Figure 7. Validation of Predicted A-to-G (or T-to-C on the - strand) Editing Sites by Sanger Sequencing in H9 and iPSC (IMR90) -4.**

(A) Editing sites in coding gene; (B) Editing sites in noncoding RNA. (C) Examination of inferred non-canonical editing sites. The sites which could not be confirmed in H9 and iPS (IMR90) -4 were labeled with blue boxes. The editing positions were highlighted by gray shading. Top and bottom panels are results from cDNA and genomic DNA respectively.


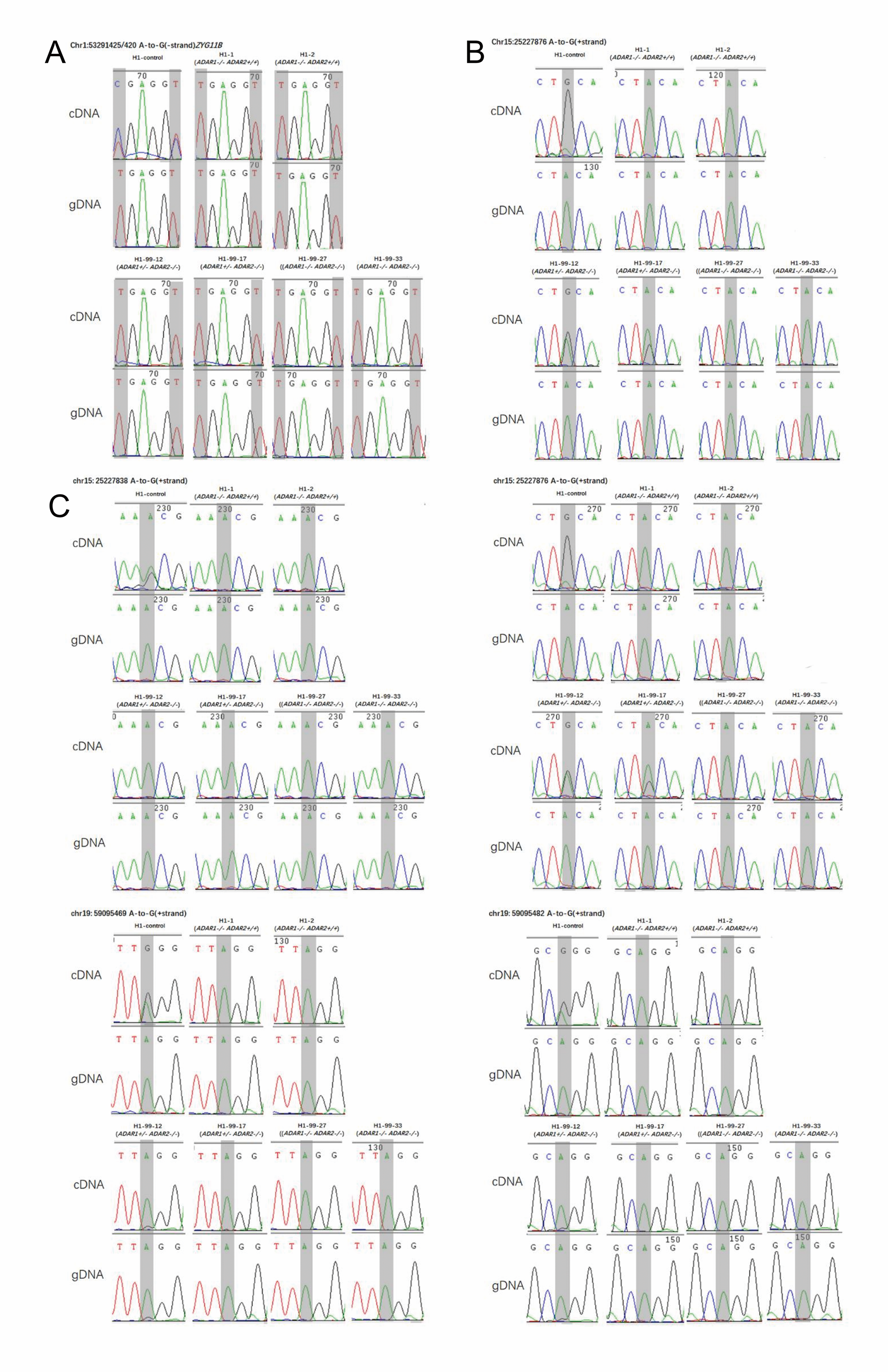


**Supplemental Figure 8. Examination of A-to-G (or T-to-C on the - strand) Editing Sites by Sanger Sequencing in *ADAR1/2* knock out H1 cell lines.**

(A) Editing site in the *ZYG11B* 420 and 425 DESs. (B) Editing site in coding gene. (C) Editing sites in noncoding RNA by Sanger Sequencing in *ADAR1/2* knock out H1 cell lines. The editing positions were highlighted by gray shading. Top and bottom panels are results from cDNA and genomic DNA respectively.

**
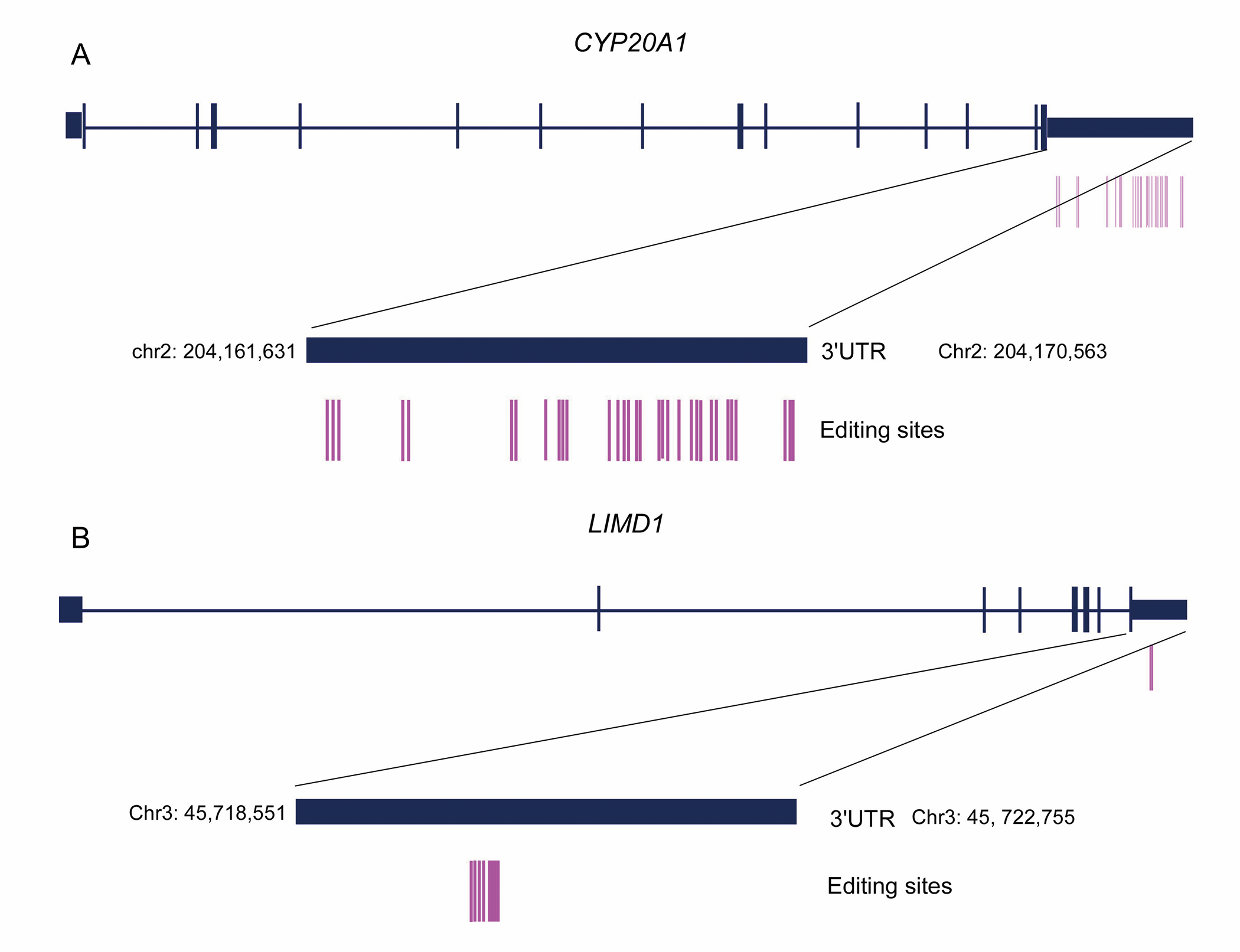
**

**Supplemental Figure 9. Distribution of RNA Editing Sites in *CYP20A1*** **(A) and *LIMD1* (B) in H1 cells**.

All 33 editing sites in *CYP20A1* and all 9 editing sites in *LIMD1* are in 3’UTR.

**
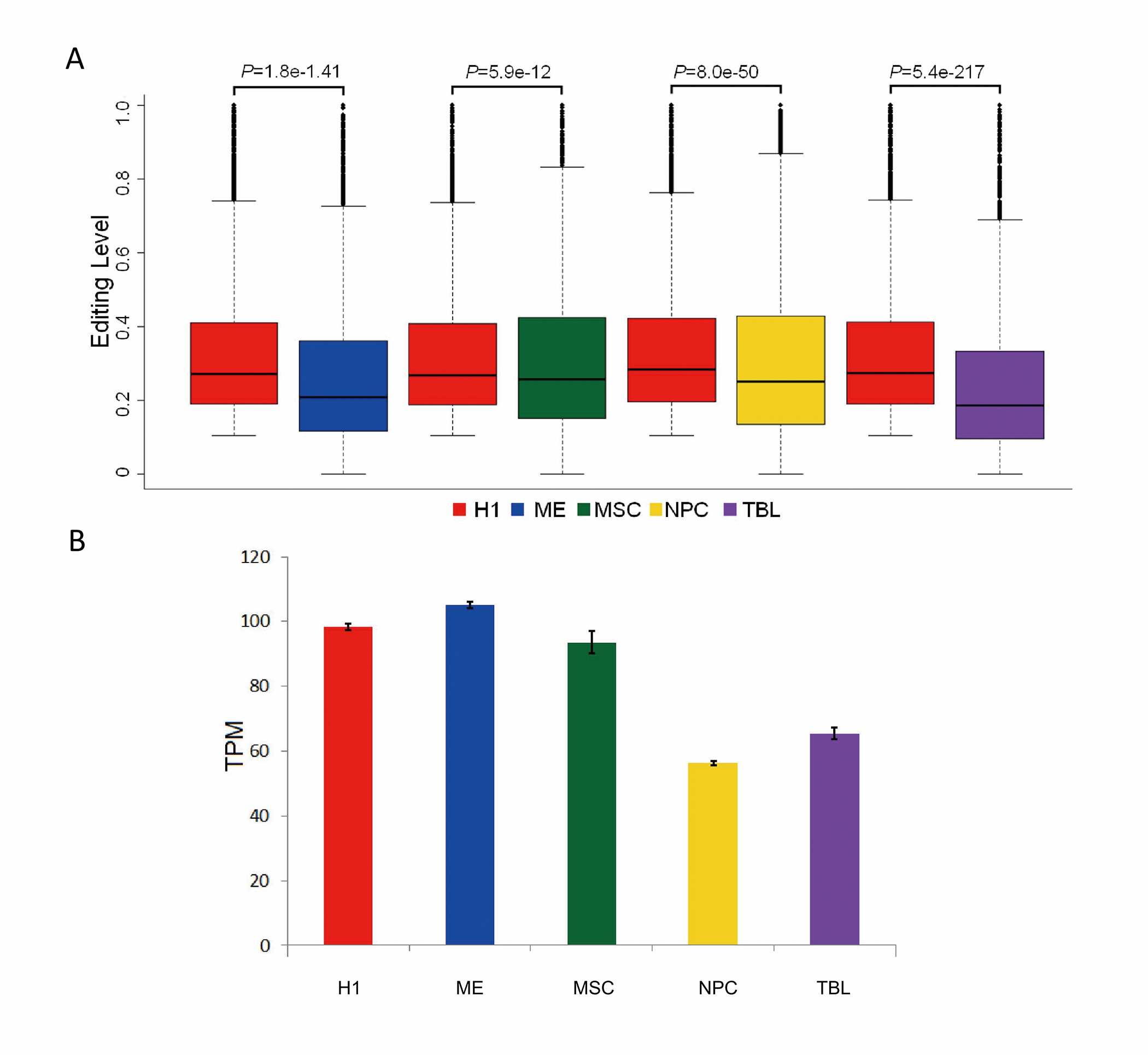
**

**Supplemental Figure 10. Pairwise comparison of RNA editing levels between H1 and its derived cells, and *ADAR1* expression in these cells.**

(A) Pairwise comparison of RNA editing levels between H1 and its derived cells. For each pair, we only include sites that are found in both cells. A Wilcoxon ranksum test was performed to decide the significant level. (B) *ADAR1* expression in H1 and its differentiated cells. The expression abundance is estimated using RSEM, and the level is measured by the number of transcripts per million reads (TPM). Error bar represents the standard deviation between two sample replicates. ME: mesendoderm cell, MSC: Mesenchymal Stem Cell, NPC: neural progenitor cell, TBL: trophoblast-like cell.


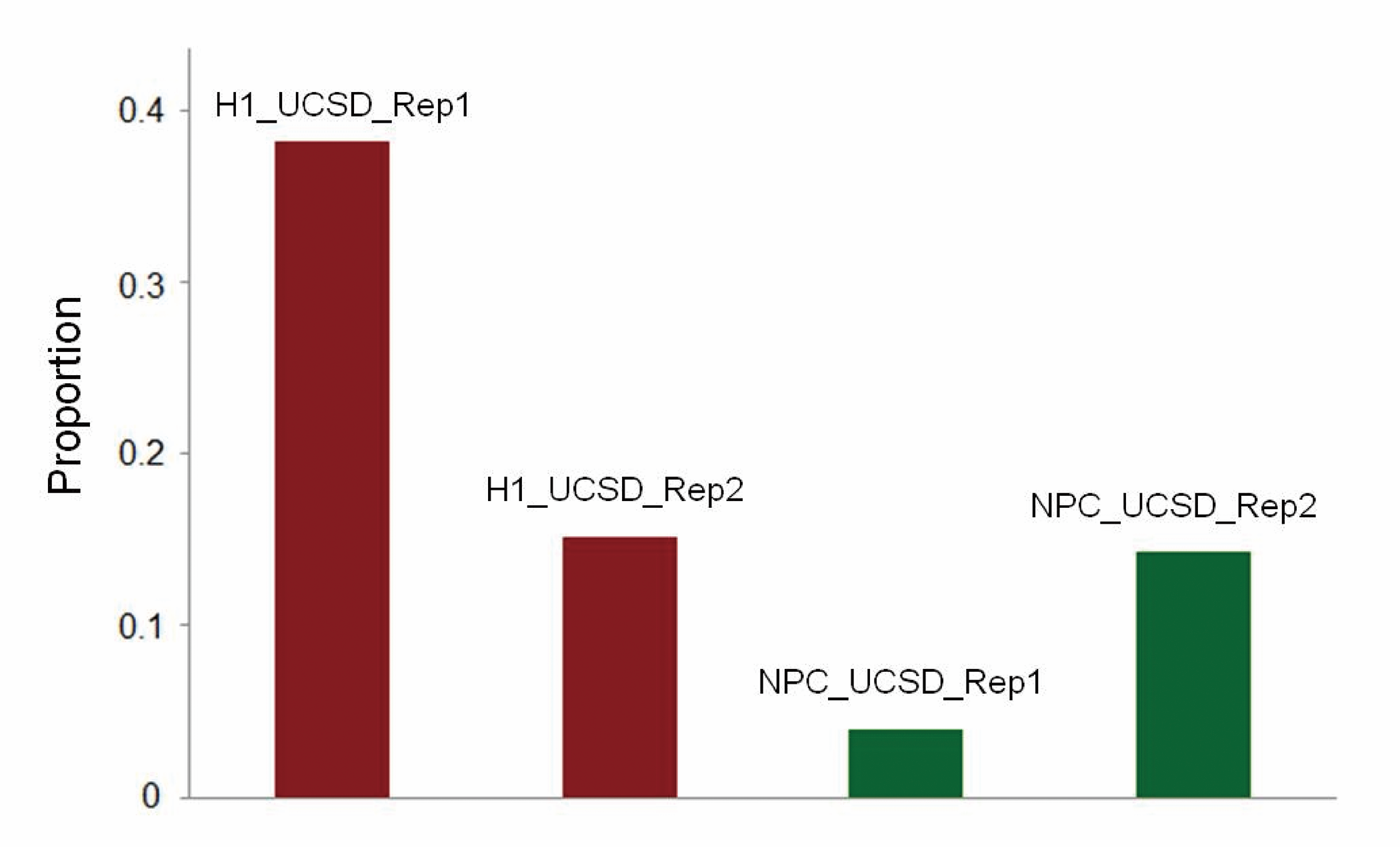


**Supplemental Figure 11. The proportion of reads with co-editing events of Chr1:53291420 and Chr1:53291425 sites in H1 and NPC replicates.**


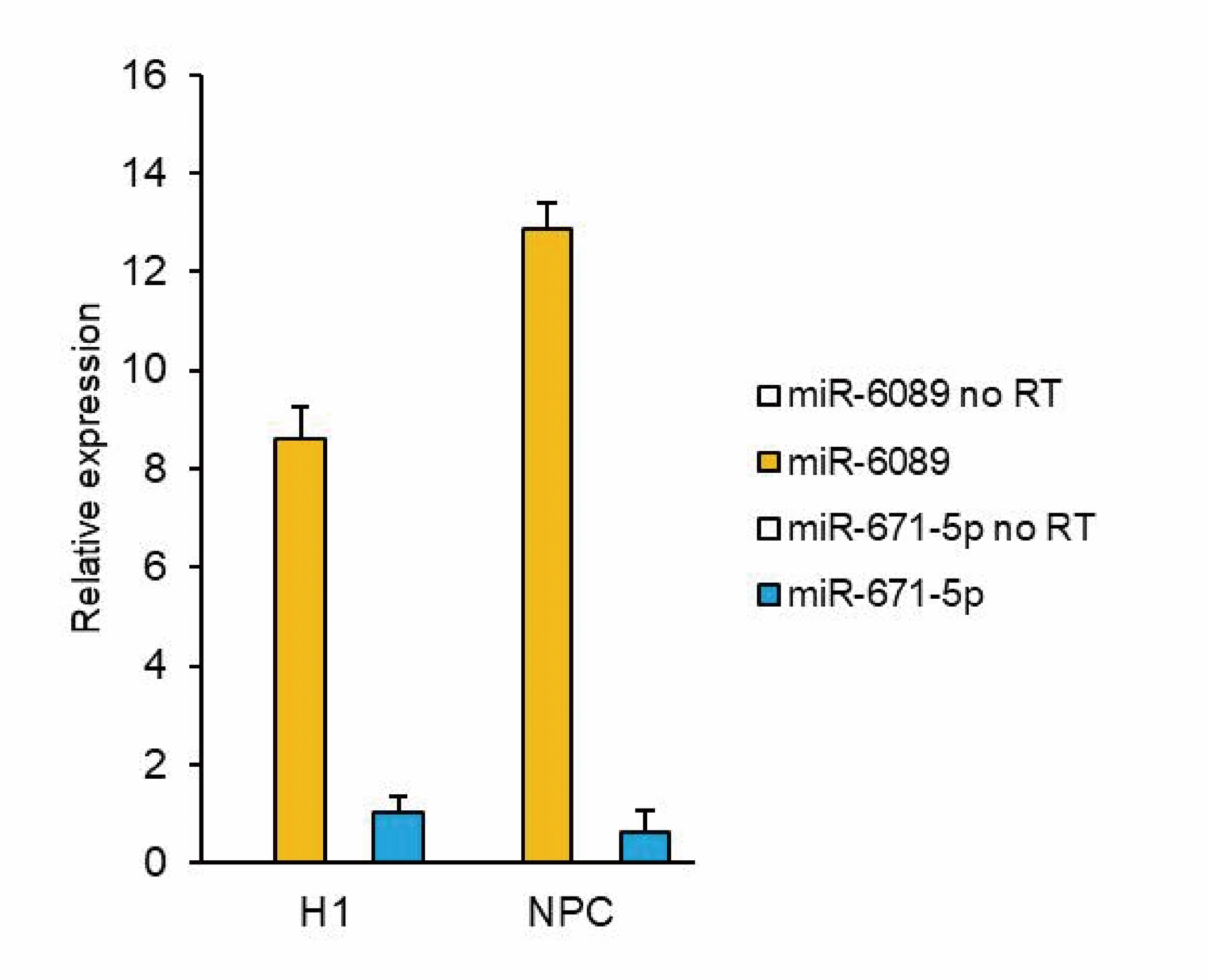


**Supplemental Figure 12. Expression of *miR-6089* and *miR-671-5p* in hESCs and differentiated NPC**.

qRT-PCR data indicated *miR-6089* and *miR-671-5p* level in hESCs and differentiated NPC at day 7. *n* = 3, (***) *P* < 0.001.


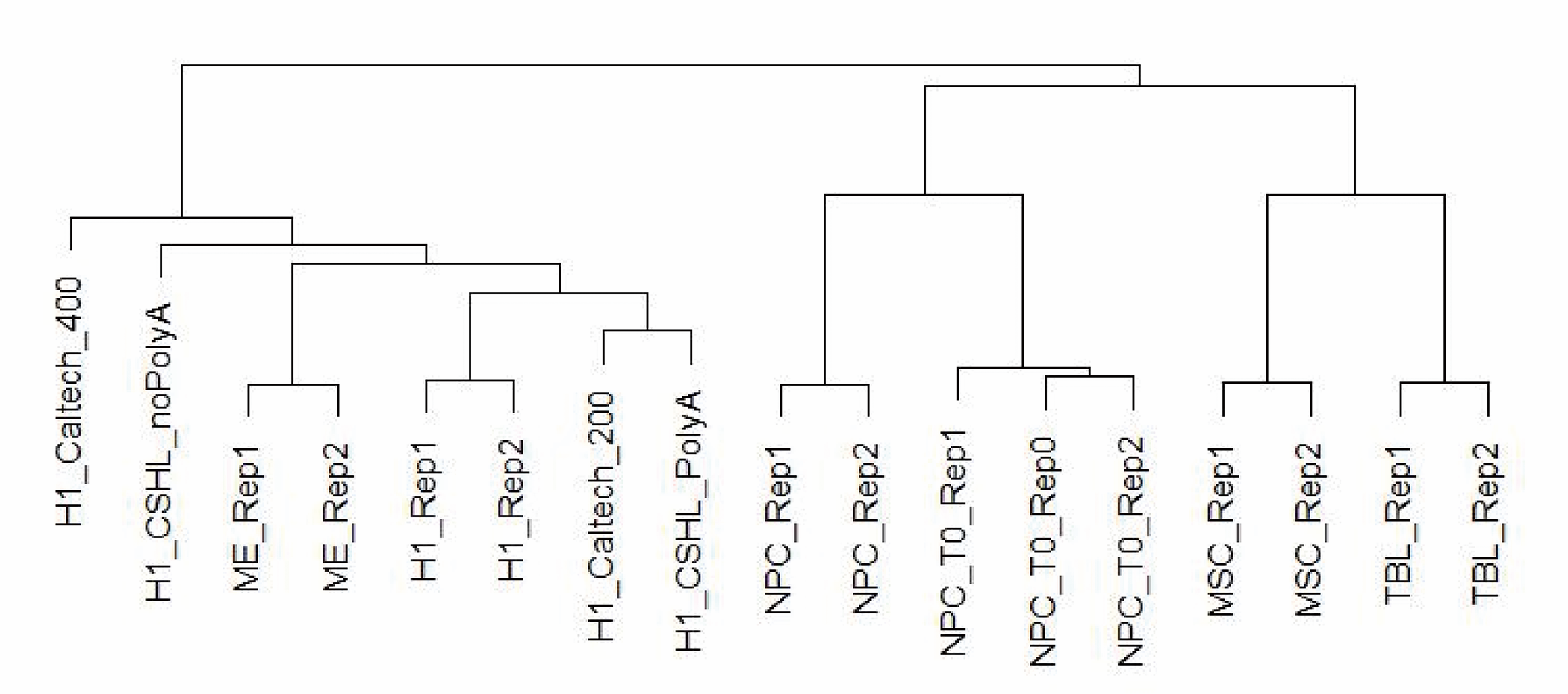


**Supplemental Figure 13. Hierarchical clustering for samples from different RNA-Seq experiments used in this study.** See Supplementary File 5 for sample information. ME : mesendoderm cell, MSC : Mesenchymal Stem Cell, NPC: neural progenitor cell, TBL : trophoblast-like cell.

Supplementary Table 1 sgRNAs used for *ADAR1/2* and the *miR6089* knock out.

| Primer name | Sequence (5’ to 3’) | Figure |
| --- | --- | --- |
| *ADAR1* sgRNA1 | GAAACTCACCTGGTGCTGCG | 1A |
| *ADAR1* sgRNA2 | TGACTTTGAAAATGGCCAGT | 1A |
| *ADAR2* sgRNA1 | GAAGTCCGTGTTGACAGACA | 1A |
| *ADAR2* sgRNA2 | GAGGACCCTGTCTGTCAACA | 1A |
| *miR6089*-sgRNA2 | CCCCTTCCGTGCGCCAGTGG | 5B |
| *miR6089*-sgRNA3 | GCGCCAGTGGAGGCCGGGGT | 5B |
| *ZYG11B3’UTR-sgRNA* | ACAGCTAAGACCAGGCACTG | 5I |

Supplementary Table 2 PCR primers used for genotyping (GT) and qPCR.

| Primer name | Sequence (5’ to 3’) | Figure |
| --- | --- | --- |
| *ADAR1*_GT_F | CCCACATCAAATGCCTCAAATAACATGG | 1A |
| *ADAR1*_GT_B | GCACTCTGTCAGTTTCTTGTAGGGTG | 1A |
| *ADAR2*_GT_F | CAATGGCCACTCCAAGTACCGCCTGAAG | 1A |
| *ADAR2*_GT_B | GAATGGTGGTAAGACAGGGAGAGGAG | 1A |
| *miR6089*-GT-1F | CAAGAAGGGCAGCGGTTTCTCTTGGCTC | 5B, S12 |
| *miR6089*-GT-1B | CGCTGGCTTTTTCCTCTGAAACGGCTC | 5B, S12 |
| *ZYG11B*-qPCR-F | TTCTGCCACCACAAGTTA | 4D, 5C, F, L |
| *ZYG11B*-qPCR-B | CCACTGATAATGTCTGTAATCG | 4D, 5C, F, L |
| *GAPDH*-qPCR-F | TGCACCACCAACTGCTTAGC | 4D, 5C |
| *GAPDH*-qPCR-B | GGCATGGACTGTGGTCATGAG | 4D, 5C |

Supplementary Table 3 PCR primers used for validating the editing sites of coding genes by Sanger sequencing.

| Coordinate | Primer name | | Sequence (5’ to 3’) | Figure |
| --- | --- | --- | --- | --- |
| Chr1:1419700/746/773 | Chr1:1419700_1F | GAAATGGAGTCGGGTTCGCTTTCTG | | S6A, S7A |
|  | Chr1:1419700_1B | CTCACGCCCGTAATCCCAGCACTTTG | | S 6A, S7A |
| ChrX:123046591/599 | ChrX:123046591_1F | GAGGCTGCATTGAGCTATGATCATGGC | | S6A, S7A |
|  | ChrX:123046591_1B | CCAACATGGTGAAACCCTGTCTCTAC | | S6A, S7A |
| Chr1:19543456/465 | Chr1:19543456_1F | GTAGCTAGGACCACAGGCACATGCCA | | 2F, S7A |
|  | Chr1:19543456_1B | CCTTGACTTACCTCTTCTCCTGTCTTC | | 2F, S7A |
| Chr15:25227876 | Chr15:25227876_1F | GTGAATGAATGAGGCAGCATTGC | | 2F, S7A, S8B |
|  | Chr15:25227876_1B | CCTACAATACCAGCTGAGGCAAGAGG | | 2F, S7A, S8B |
| Chr1:53291425/420 | Chr1:53291425_1F | GGTTGAGGTGAATAAAGCTGCATGGG | | 4B, 5K, S8A |
|  | Chr1:53291425_1B | GAAGATGGCCCGGTGTTATGTTGATG | | 4B, 5K, S8A |

Supplementary Table 4 PCR primers used for validating non-canonical editing sites by Sanger sequencing.

| Coordinate | Primer name | Sequence (5’ to 3’) | Supplementary figure |
| --- | --- | --- | --- |
| Chr19:54377543 | Chr19:54377543_1F | GTTCATCAGCGACCCCAACCTGTAC | S6C, S7C |
|  | Chr19:54377543_1B | GTCAGCCACATACGCCAGTAGGTTG | S6C, S7C |
| Chr2:48603789 | Chr2:48603789_1F | GAGGAGGCAGTGTAACAACCTATAGTGC | S6C, S7C |
|  | Chr2:48603789_1B | GACTTAGGATGTAGCATAAGACTGAAC | S6C, S7C |
| Chr3:12939244 | Chr3:12939244_1F | CTGCTCAGTGTTGCAATGCTGCCTTG | S6C, S7C |
|  | Chr3:12939244_1B | GTCTTCAGCACCCAGTGCAGTGATC | S6C, S7C |
| Chr7:28998010 | Chr7:28998010_1F | GCAGACCCCGGGTACTACTTGTTGC | S6C, S7C |
|  | Chr7:28998010_1B | AAGCTCTTGGAACGGCTCCCGCCTGCA | S6C, S7C |

Supplementary Table 5 PCR primers used for validating editing sites in lncRNA by Sanger sequencing.

| Coordinate | Primer name | Sequence (5’ to 3’) | Supplementary figure |
| --- | --- | --- | --- |
| Chr1:19620137/461/487/654 | Chr1:19620137_1F | GCACCATGACCAAGTTAGTTTCATCCCAG | S6B, S7B |
|  | Chr1:19620137_1B: | CAGAACCCAGAAGCCTGACATGCTGTC | S6B, S7B |
| Chr15:25227838/7876/8815 | Chr15:25227838_1F: | GGAACACAGTGAGTATAGTACCTGCC | S6B, S7B, S8C |
|  | Chr15:25227838_1B: | AGTACCTGGCACAGTGGCTCACACCTAC | S6B, S7B, S8C |
| Chr7:130629624 | Chr7:130629624_1F: | TGGCGCCAGTGACAGGAATCAGTTCTG | 3G, S7B |
|  | Chr7:130629624_1B: | CTGAAGTCTAGGCAAGTGGCTGAGCTAGG | 2G, S7B |
| Chr1:20976013/023/032/095 | Chr1:20976013_1F | GTAGGTAGGAGTAGGAGCACTAGCCACC | S6B, S7B |
|  | Chr1:20976013_1B | GACTTGCACTGTCACCTAGGCCAGAG | S6B, S7B |
| Chr15:40986496/505 | Chr15:40986496_1F | CGAGTAGCTAGGATTACAGGCATGTG | 2G, S6B, S7B |
|  | Chr15:40986496_1B | GCTGGAATATTCGTCAATCTGTGGTGC | 2G, S6B, S7B |
| Chr19:59095469/482 | Chr19:59095469_1F | GCTGTCCATCATGGGTATATCTCTGG | 2G, S6B |
|  | Chr19:59095469_1B | CTTCAGTCTCTTGCCTTGTCACCTGC | 2G, S6B |
| Chr3:38493029/030 | Chr3:38493029_1F | GTCACCTGAAGTTGGGAGTTCAAGAC | S7B |
|  | Chr3:38493029_1B | CCTTATGAGTCCTACTGAAACCAGCC | S7B |
